# Inflammation and blood-brain barrier breach remote from the primary injury following neurotrauma

**DOI:** 10.1186/s12974-018-1227-0

**Published:** 2018-07-07

**Authors:** Nicole M. Smith, Marcus K. Giacci, Alexander Gough, Charlotte Bailey, Terence McGonigle, Anna M. B. Black, Thomas O. Clarke, Carole A. Bartlett, K. Swaminathan Iyer, Sarah A. Dunlop, Melinda Fitzgerald

**Affiliations:** 1 School of Molecular Sciences, The University of Western Australia, Stirling Hwy, Perth, Western Australia 6009 Australia; 2 Experimental and Regenerative Neurosciences, School of Biological Sciences, The University of Western Australia, Stirling Hwy, Perth, Western Australia 6009 Australia; 3 Curtin Health Innovation Research Institute, Curtin University, Verdun St, Nedlands, Western Australia Australia; 4 Perron Institute for Neurological and Translational Science, Sarich Neuroscience Research Institute, Verdun St, Nedlands, Western Australia 6009 Australia

**Keywords:** Neurotrauma, Secondary degeneration, Microglia, Macrophages, Cytokines, Blood-brain barrier

## Abstract

**Background:**

Following injury to the central nervous system, increased microglia, secretion of pro- and anti-inflammatory cytokines, and altered blood-brain barrier permeability, a hallmark of degeneration, are observed at and immediately adjacent to the injury site. However, few studies investigate how regions remote from the primary injury could also suffer from inflammation and secondary degeneration.

**Methods:**

Adult female Piebald-Viral-Glaxo (PVG) rats underwent partial transection of the right optic nerve, with normal, age-matched, unoperated animals as controls. Perfusion-fixed brains and right optic nerves were harvested for immunohistochemical assessment of inflammatory markers and blood-brain barrier integrity; fresh-frozen brains were used for multiplex cytokine analysis.

**Results:**

Immediately ventral to the optic nerve injury, immunointensity of both the pro-inflammatory biomarker inducible nitric oxide synthase (iNOS) and the anti-inflammatory biomarker arginase-1 (Arg1) increased at 7 days post-injury, with colocalization of iNOS and Arg1 immunoreactivity within individual cells. CD11b+ and CD45+ cells were increased 7 days post-injury, with altered BBB permeability still evident at this time. In the lower and middle optic tract and superior colliculus, IBA1+ resident microglia were first increased at 3 days; ED1+ and CD11b+ cells were first increased in the middle and upper tract and superior colliculus 7 days post-injury. Increased fibrinogen immunoreactivity indicative of altered BBB permeability was first observed in the contralateral upper tract at 3 days and middle tract at 7 days post-injury. Multiplex cytokine analysis of brain homogenates indicated significant increases in the pro-inflammatory cytokines, IL-2 and TNFα, and anti-inflammatory cytokine IL-10 1 day post-injury, decreasing to control levels at 3 days for TNFα and 7 days for IL-2. IL-10 was significantly elevated at 1 and 7 days post-injury with a dip at 3 days post-injury.

**Conclusions:**

Partial injury to the optic nerve induces a complex remote inflammatory response, characterized by rapidly increased pro- and anti-inflammatory cytokines in brain homogenates, increased numbers of IBA1+ cells throughout the visual pathways, and increased CD11b+ and ED1+ inflammatory cells, particularly towards the synaptic terminals. BBB permeability can increase prior to inflammatory cell infiltration, dependent on the brain region.

## Background

Following an injury to a white matter tract in the central nervous system (CNS), axons located at the primary injury site are lesioned and degenerate [1]. Neurons and glia spatially separated from the site of initial injury to axons undergo further damage and activation due to secondary degeneration [2]. Inflammation following CNS trauma is responsible for both beneficial and detrimental effects, contributing to secondary damage but also facilitating neurorepair [3]. Activation of resident microglia, secretion of immune mediators such as pro- and anti-inflammatory cytokines/chemokines, and infiltration of blood leukocytes characterize this robust inflammatory response [4]. The release of reactive oxygen species (ROS) is a key pro-inflammatory feature, leading to glial activation and oxidative damage in the tissue surrounding the initial injury site [5] early after injury [6, 7]. Protective anti-inflammatory responses include myelin debris phagocytosis [8, 9] and neurotrophic and growth factor secretion [10]. Astrocyte activation leads to a breach of the blood-brain barrier (BBB), with increased monocyte extravasation [11] and associated increases in Ca^2+^ and oxidative cascades of injury to both axons and glia, further contributing to spreading of degeneration [12].

Because macrophages and microglia are key modulator and effector cells in the immune response, much attention has been devoted to defining the dynamics of pro- and anti-inflammatory effects of trauma-induced inflammation within the linear constraints of microglia/macrophage polarization phenotypes, namely anti-inflammatory “M1” vs pro-inflammatory “M2” [5, 13–17]. The in vivo environment is complex and presents a variety of pro- and anti-inflammatory stimuli simultaneously [18–20]. In fact, recent in vivo studies of inflammation following CNS trauma have highlighted the plasticity of these cells where the same cell population is able to switch between polarization states in response to mediators in their surrounding microenvironment, and yet, others report the concurrent expression of both pro- and anti-inflammatory markers by a single cell [21–24]. These findings suggest a lack of exclusivity in microglia/ macrophage activation states in vivo and indicate that the same cell responsible for promoting neuroinflammation can also release factors that promote wound healing. It is likely that the combination and balance of multiple factors (i.e., glial cells, immune cells, pro- and anti-inflammatory cytokines/ chemokine levels, time elapsed after injury, and lesion magnitude) will lead to different degrees of damage, as has been described in a range of injury models including glaucomatous damage at the optic nerve head, partial optic nerve transection, and spared nerve injury [25–27]. Furthermore, it is increasingly understood that specific monocyte, macrophage, and other glial and endothelial cell-derived cytokines play important roles in maintaining or perturbing BBB permeability, thereby further modulating the injury response [28]. Characterizing the magnitude, timing, and duration of expression of these mediators has the potential to offer mechanistic insight and aid in the development of novel therapeutics.

Despite the plethora of research on trauma-induced inflammation at the site of a primary injury and immediately adjacent, relatively few studies investigate how regions remote from the primary injury site could also suffer from secondary degeneration and inflammation following neurotrauma [29–31]. Clinical studies suggest a link between remote damage and symptomatology, a phenomenon termed “diaschisis,” which contributes to functional impairments [32–34]. Previous studies in the spinal cord have shown that remote injury induces substantial changes in microglia and astrocytes, which contribute to central inflammatory responses [35, 36]. In addition, peripheral nerve injury evokes trafficking of immune cells from the circulation into the spinal cord parenchyma [37]. Using our in vivo partial optic nerve transection model of secondary degeneration in adult Piebald-Viral-Glaxo (PVG) female hooded rats, we have previously demonstrated that a remote injury in the CNS (optic nerve) can cause a transient opening of the BBB in the visual pathways of the brain as early as 1 day following injury, with Evans blue fluorescence visible around the optic chiasm, optic tract, lateral geniculate nucleus, and superior colliculus. BBB permeability was maximal at 3 days following injury and decreased to levels not significantly different from uninjured controls at 7 days [38]. An initial analysis of the inflammatory response early (1 day) following injury indicated that ED1+ and IBA1+ microglia/macrophage activation occurred along the length of the injured optic nerve, in the optic chiasm, and adjacent to the cerebral ventricles [38], and earlier studies have shown increases in these cells immediately adjacent to the injury at 3 days [25]. However, a quantitative assessment of a range of inflammatory cell subtypes along the optic tract and into the superior colliculus is lacking. Furthermore, the spatiotemporal relationship between inflammation and blood-brain barrier breach remote to an injury is unknown. Specifically, it is not yet clear whether inflammatory cells that rapidly infiltrated the injury site spread along the optic nerve and tract into the brain, thereby initiating BBB breakdown. Alternative hypotheses include axonal degeneration and compromise of axonal transport initiating remote inflammation and BBB breakdown close to synaptic terminals in the superior colliculus or systemic changes contributing to a widespread inflammatory response. Here, we assess microglia and macrophage dynamics together with indicators of BBB breach and cytokine profiles, at various sites distal to a partial optic nerve transection injury, focusing on identifying the time at which changes are first observed in order to determine in a region-specific manner, whether blood-brain barrier breach precedes inflammation.

## Methods

### Animal procedures

Adult female PVG hooded rats (160–180 g) were bred at the Animal Resources Centre (Murdoch, WA, Australia), housed under a standard 12-h light/dark cycle and fed standard rat chow and water ad libitum. Procedures adhered to the National Health and Medical Research Council Australian Code of Practice for the care and use of animals for scientific purposes and were approved by the University of Western Australia’s Animal Ethics Committee, approval numbers RA3/100/1201, RA3/100/673, and RA3/100/1485. The rats were anesthetized intraperitoneally with a combination of xylazine (Ilium Xylazil, Troy Laboratories, 10 mg/kg) and ketamine (Ketamil, Troy Laboratories, 50 mg/kg), and euthanized with an intraperitoneal injection of Euthal (pentobarbitone sodium 850 mg/kg; phenytoin sodium 125 mg/kg; Virbac).

The partial optic nerve transection procedure was similar to that described previously [25]. Briefly, anesthetized rats were shaved along the skull towards the right eye. Under aseptic conditions, the skin was incised and retracted, and the right optic nerve accessed by deflecting the Harderian lachrymal gland immediately behind the right eye. The nerve parenchyma was exposed about 1 mm behind the eye by making a slit in the dura mater with ophthalmic scissors. A controlled 200-μm cut was made in the dorsum of the right optic nerve using a diamond radial keratotomy knife (Geuder), with the depth determined by the protrusion of the blade beyond a surrounding guard. Care was taken not to stretch the optic nerve or damage major ophthalmic blood vessels. The deflected tissue was replaced, and the skin was sutured with 4/0 non-absorbable silk suture. The eyes were lubricated with Luxyal eye drops, and the rats were placed on a warming blanket for recovery with subcutaneous injections of analgesic (Carprieve, 5 mg kg^−1^ in sterile water, Norbrook Australia, Pty. Ltd.; Victoria, Australia) and sterile PBS (1 ml). No postoperative infections were observed.

The right optic nerves from normal, age-matched, unoperated animals were used as controls, as sham-operated animals have been shown to be not different to normal in terms of a range of relevant cellular and oxidative stress outcomes [25]. Three cohorts of animals were used, with animals within each cohort being randomized into groups reflecting the number of days post-injury, for details see Table 1. In brief, cohort 1 was used for immunohistochemical studies assessing microglia/macrophages in the right optic nerves of animals at 3, 7, and 28 days post-injury and in uninjured controls. For cohort 1, three groups of uninjured controls collected at days 3, 7, and 28 were compared and found to be not different (ANOVA, *p* > 0.05); the 3-day controls were used for statistical comparisons. Cohort 2 was used for BBB analyses in the optic tracts and superior colliculus of animals at 3, 7, 14, and 28 days post-injury and in uninjured controls, assessing the presence of Evans blue dye. Once it was established that no Evans blue dye was visible in the optic tracts and superior colliculus, a subset of cohort 2 was randomly selected for immunohistochemical studies assessing microglia and macrophages as well as fibrinogen and caveolin-1 immunoreactivity with increases being surrogate markers for BBB breach in these brain regions (*n* = 5–6/group). Cohort 3 was used for cytokine analyses in the homogenized brains of animals at 1, 3, and 7 days post-injury and in uninjured controls.

**Table 1:** Summary of cohorts and numbers of animals per group

	Uninjured	Day 1	Day 3	Day 7	Day 14	Day 28
Cohort 1						
- Microglia/macrophages in the optic nerve	*n* = 10*		*n* = 10	*n* = 10		*n* = 10
Cohort 2						
- BBB analyses	*n* = 9–12 EB		*n* = 9–12 EB	*n* = 9–12 EB	*n* = 9–12 EB	*n* = 9–12 EB
- Microglia/macrophages in the optic tract	*n* = 9	*n* = 6 no EB	*n* = 6	*n* = 5–6	*n* = 4–5	*n* = 4
Cohort 3						
- Cytokines	*n* = 8	*n* = 8	*n* = 8	*n* = 8		

Day is the day after partial optic nerve transection injury. * For cohort 1, three groups of uninjured controls collected at days 3, 7, and 28 were compared and found to be not different (*p* ≤ 0.05); the 3-day controls were used for statistical comparisons. EB denotes intravenous injection of Evans blue for the specified animals. Numbers of animals per group are presented as a range if suitable sections were not available for specific brain regions. BBB analyses encompass assessments of Evans blue, caveolin, and fibrinogen immunoreactivity

### Immunohistochemistry, microscopy and analysis

Animals for BBB analyses (cohort 2) were administered a 2% (*w*/*v*) sterile solution of Evans blue (Sigma, E2129_10G) in PBS (2.8 ml/kg) via tail vein injection 1 h prior to euthanasia. Immediately following euthanasia, the animals for cohorts 1 and 2 (BBB and immunohistochemical assessments) were transcardially perfused-fixed with 0.9% (*w*/*v*) saline followed by 4% (*w*/*v*) paraformaldehyde (Sigma-Aldrich; St. Louis, Missouri, USA) in 0.1 M phosphate buffer, pH 7.2–7.4. The right optic nerves and brains were harvested and post-fixed in 4% paraformaldehyde for 1 h (nerves) or overnight (brains) and then cryoprotected in 15% sucrose in PBS and stored at 4 °C. The optic nerves were cryosectioned transversely (14 μm thickness), and the brains were cryosectioned coronally (20 μm thickness) and collected onto a series of slides so that each slide had a representative section from each area of interest. Sections for Evans blue analysis were collected onto Superfrost® Plus slides, air dried for an hour, and only briefly rinsed in PBS to reduce leakage of the dye from the sections before mounting using Fluoromount-G (Southern Biotech). The sections for immunohistochemistry were collected onto Superfrost® Plus slides and stored at − 80 °C.

Immunohistochemical analyses were conducted according to the previously described procedures [39]. Primary antibodies used for immunohistochemical assessments of the optic nerves detected inducible nitric oxide synthase (iNOS) (1:400, BD Biosciences, ab3523), Arginase-1 (Arg-1) (1:400, Santa Cruz Biotechnology, sc-18355), CD45 (1:200, BD Pharmingen, 555482), and CD11b (1:400, 553310, BD Pharmingen). Primary antibodies used for immunohistochemical assessments of the brains (optic tracts and superior colliculus) recognized CD45 (1:500, Abcam, ab10558), CD11b (1:300, Sapphire Bioscience, ab2910), ionized calcium-binding adapter molecule 1 (IBA1) (1:1000, Abcam, ab5076), CD68 (ED1) (1:1000, Millipore Corporation, MAB1435), Caveolin-1 (1:400, Sapphire Bioscience, Ab2910), and Fibrinogen (1:500, HiTech Group Australia, A008002). Secondary antibodies were species-specific AlexaFluor 488-, 555-, and 647-conjugated antibodies (1:500, Invitrogen Australia). All tissue sections for a particular immunohistochemical outcome were processed at the same time.

Fluorescence imaging was performed using a Nikon Si1 inverted microscope (Nikon Corporation, Japan) for the right optic nerve and optic tract assessments of microglia and macrophages and a Leitz Diaplan microscope with an Olympus DP70 camera controlled by DP controller (version 2.1.1.183) for superior colliculus; a confocal Nikon C2 mounted on an upright Ni-E microscope, for assessments of BBB integrity (Evans blue fluorescence, fibrinogen, and caveolin-1 immunoreactivity), or for immunohistochemical assessments of microglia and macrophages in the brain (optic tracts and superior colliculus). Images from the Nikon microscopes were captured in a stack of 13 optical slices at 0.5 μm increments along the *z*-axis, obtained from the middle 6 μm of the section, controlled by NIS elements 4.3 software. All images for assessment of each outcome measure were captured at a constant exposure, using consistent microscope settings.

**Fig. 1:**
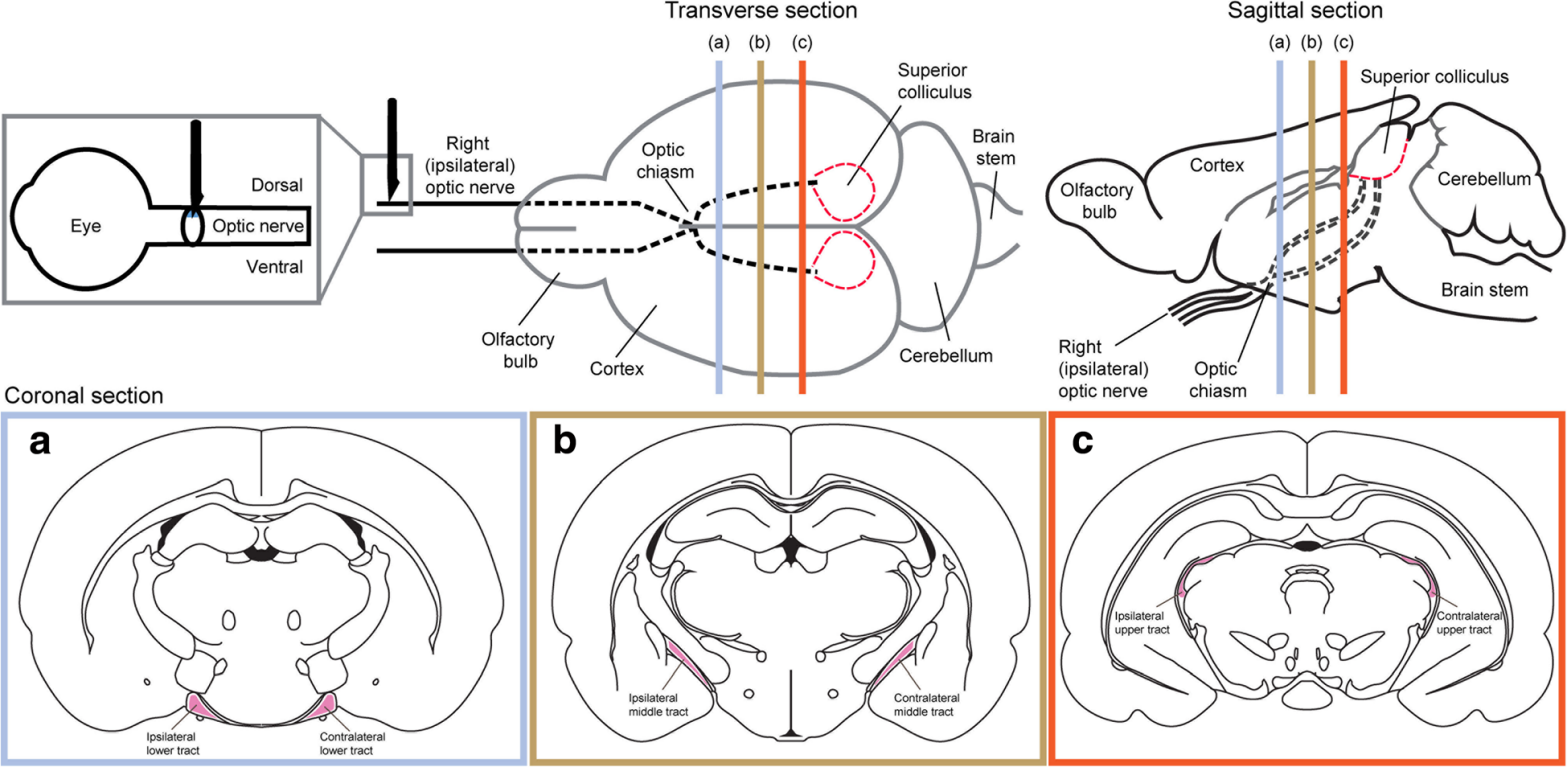
Schematic overview of the regions of interest used for quantification of the inflammatory response following partial injury to the optic nerve. The ventral aspect of the optic nerve (gray box) was used to quantify Arg1 and iNOS immunoreactivity and CD11b and CD45 cell density. The lower (blue box), middle (yellow box), and upper (red box) tracts were used to quantify the density of CD11b-, IBA1-, ED1-, CD45-, and CD11b/IBA1/ED1-positive cells

A single section was selected for optic nerves that showed the dorsal injury site and the ventral nerve. The fields of view imaged were located in the ventral portion of the right optic nerve of that section, directly below the dorsal primary injury site. The intensities of iNOS and Arg-1 immunoreactivity were assessed on the single central optical slice most in focus from the relevant 13 image z-stack. Mean fluorescence intensities were quantified on the whole image of ventral optic nerve and were normalized by subtracting the average of three randomly selected background regions of interest per animal. The numbers of CD45+ and CD11b+ cells were assessed within similar regions of interest. This non-stereological technique does not allow for an estimate of total cell numbers within the nerve but does allow for comparative assessment between time points after injury. Similarly for the brains, assessments of the numbers of CD45+, CD11b+, IBA1+, and ED1+ cells were conducted in single visual slices from images of single sections capturing either the lower, middle, or upper regions of both the ipsilateral and contralateral optic tracts and at the rostral end of the superior colliculus for each animal (Fig. 1). While sampling of more than one section may have been preferable, the brain regions of interest were often only clearly present in one section per slide. The optic tract is relatively small and was only apparent in one section per region of interest per slide along the rostrocaudal plane. Similarly, the optic nerve injury site, and therefore the ventral region directly under the injury, is only clearly apparent in one or two sections per slide. Not all markers were assessed for all regions at all time points; analyses were based on identifying the first time at which significant increases were observed and at times were constrained by the availability of sections containing the precise region of interest (e.g., superior colliculus at day 1). The numbers of positively stained cells within each field of view were expressed per unit area. Assessments of Evans blue fluorescence were conducted qualitatively, scanning multiple sections of the right optic nerves and regions throughout the brain including optic tracts, ventricles, choroid plexus, and the superior colliculus and applying a score to fluorescence; representative images are shown. Caveolin-1 immunoreactivity of endothelial cells lining the ventricles in the brain was quantified by measuring the length of immunoreactive cells expressed as a percentage of the total length of the lining of the ventricle that was visible in the single image used for analysis; the choice of section for imaging was based upon ventricle size and shape to ensure consistency of the region of analysis for all animals. Assessment of fibrinogen immunofluorescence was performed throughout the optic tracts, on single central visual slices from the relevant 13 image z-stack. Fibrinogen was quantified as mean fluorescent intensity and the area above the threshold as a percentage of the total area of interest. Threshold values were normalized to the background of each image by dividing the average of three random background measurements by the average background of a control image then multiplying the original threshold value by this transformation factor. Average intensities above the threshold were normalized by subtracting the background from the mean fluorescence intensity.

### Cytokine measurements—multiplex assay

Rats from cohort 3 (*n* = 8 per group) were euthanized and transcardially perfused with ice-cold 0.9% saline, after which the brains were harvested, washed with ice-cold PBS, and homogenized in ice-cold RIPA buffer (Sigma Aldrich) supplemented with 1× protease inhibitor cocktail (Sigma Aldrich) (10 ml). Brain homogenates were incubated on ice for 20 min to aid protein solubilization, after which they were centrifuged at 12000*g* for 20 min at 4 °C, and the lysates were stored at − 80 °C until use. Total protein concentration of the brain lysates from each animal was determined using the Pierce BCA Protein Assay Kit (Thermo Fisher Scientific Pty Ltd.) according to the manufacturer’s instructions.

The concentration of ten cytokines (GM-CSF, Interleukin (IL)-12, Interferon (IFN)-γ, IL-1α, IL-1β, IL-2, IL-4, IL-6, IL-10, tumor necrosis factor (TNF)-α) in brain lysates was determined using the Cytokine Rat Magnetic 10-Plex Panel kit (Life Technologies) according to the manufacturer’s instructions using the Luminex® 200™ system (CMCA). Briefly, brain lysates were thawed, clarified by centrifugation (1000*g* for 10 min at 4 °C), and standardized at 500 μg/ml protein using assay diluent provided in the Rat Cytokine 10-Plex kit. Lyophilized cytokine standards (Rat Cytokine 10-Plex kit) were reconstituted in assay diluent (Rat Cytokine 10-Plex kit), and 1:3 serial dilutions were performed with assay diluent as instructed (Rat Cytokine 10-Plex kit) to give seven standard solutions. Colored magnetic beads conjugated with cytokine antibodies (Rat Cytokine 10-Plex kit) were reconstituted in 1× wash solution (Rat Cytokine 10-Plex kit) to give a 1× antibody bead solution, which was then pipetted into 80 wells of a 96-well plate (25 μl/well). The antibody beads were washed twice with 1× wash solution (200 μl/well). Following washing, prepared incubation buffer (Rat Cytokine 10-Plex kit) was added to each of the 80 wells (50 μl/well). Seven cytokine standard solutions were added in duplicate to wells designated for standards (100 ul/well), assay diluent was added in duplicate to wells designated for blanks (100 μl/well), and the 32 brain lysate samples from each animal (500 μg/ml) were added in duplicate to wells designated for samples (50 μl of sample + 50 μl of assay diluent), following which the plate was incubated at room temperature on a shaking platform (2 h) to enable cytokines to bind to their respective antibody beads. Following incubation, the wells were washed twice with 1× wash solution (200 μl/well), and subsequently, a 1× biotinylated antibody solution (Rat Cytokine 10-Plex kit) containing a mixture of biotinylated antibodies against each cytokine was added to each of the 80 wells (100 μl/well) and incubated at room temperature on a shaking platform (1 h). Following incubation, the wells were washed twice with 1× wash solution (200 μl/well), and subsequently, a 1× streptavidin-phycoerythrin solution (Rat Cytokine 10-Plex kit) was added to each of the 80 wells (100 μl/well) and incubated at room temperature on a shaking platform (30 min). Following incubation, the wells were washed thrice with 1× wash solution (200 μl/well) and stored in 1× wash solution (125 μl/well) overnight at 4 °C. The following day, working wash solution was decanted, and fresh 1× working wash solution was added (125 μl/well). The plate was then placed on a shaking platform (3 min) and inserted into an XY platform on the Luminex® 200™ system (CMCA) to measure cytokine concentrations for each sample calculated against the standard curve. Each well was sampled using a volume of 75 μl and a bead count of 100 per analyte before advancing to the next well.

### Data analysis and statistics

Data are presented as mean ± standard error of mean. Statistical analyses of activated microglia and macrophage densities, Arg1 and iNOS immunointensities, and caveolin-1 and fibrinogen immunoreactivity were performed on SPSS (IBM) software, using one-way ANOVA or two-way ANOVAs to compare regions at a particular time point. For two-way ANOVAs, overall *p*, *F*, and df values are provided, with *p*, *F*, and df values provided for subsets where significance was reached. Tukey post hoc tests were used for optic nerve data and Sidak post hoc tests for optic tract data. If Levene’s test indicated inequality of variances, data were log or square root transformed for two-way ANOVAs or Games-Howell post hoc tests employed for one-way ANOVAs. Post hoc *p* values were provided where significance was reached using ANOVAs. Statistical analyses of cytokine data were performed on GraphPad Prism 6 software using one-way ANOVA to compare means of *n* = 8 animals at each time point, conducted individually for each cytokine, using Tukey post hoc tests.

## Results

### Inflammatory responses in optic nerve immediately adjacent to the primary injury

**Fig. 2:**
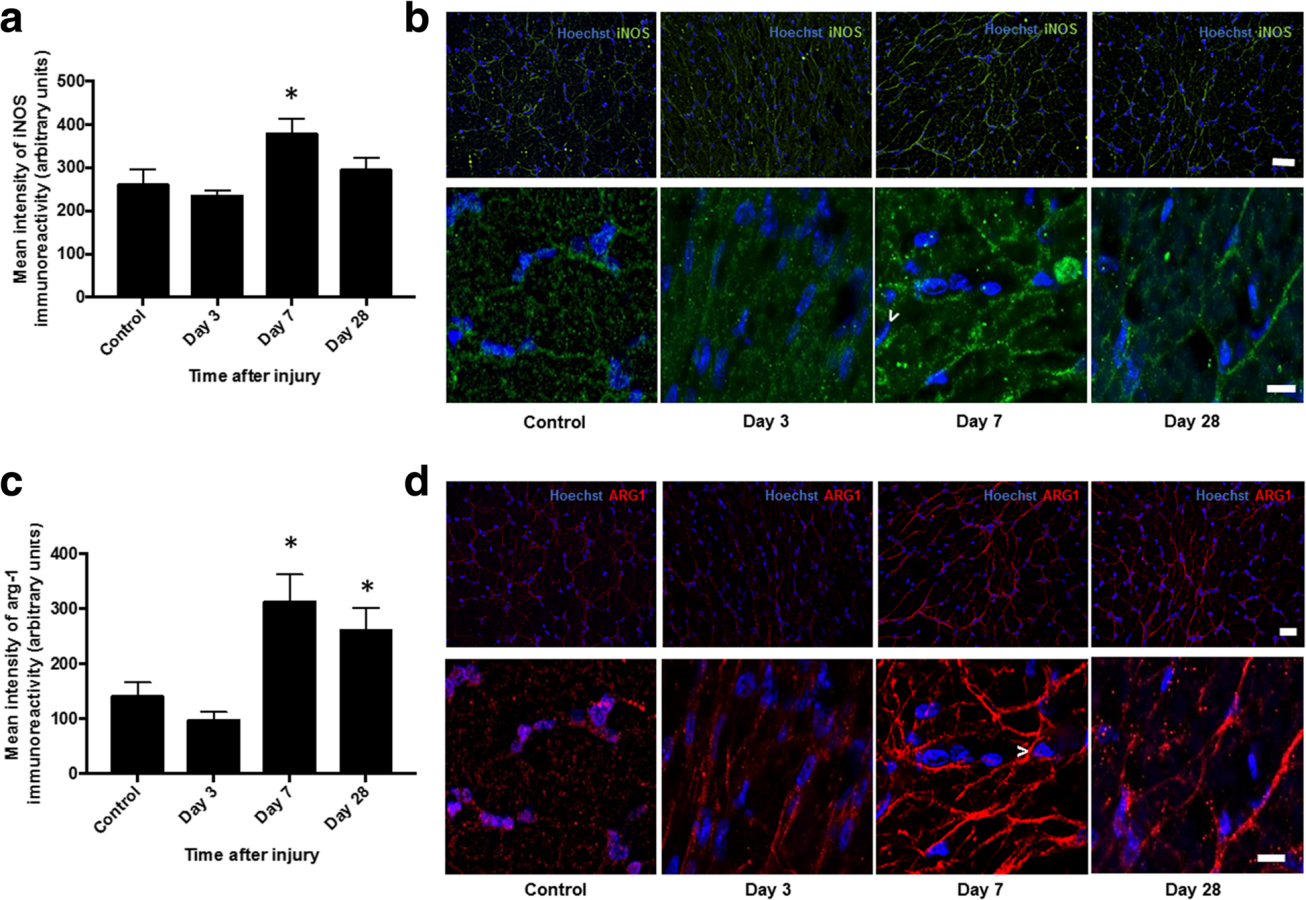
Semi-quantification of iNOS and Arg1 immunoreactivity in ventral ON vulnerable to secondary degeneration. Mean ± SEM fluorescence intensity of **a** iNOS and **c** Arg1 in the control optic nerve and at 3, 7, and 28 days following partial optic nerve transection, in ventral optic nerve vulnerable to secondary degeneration are shown. * indicates significantly different from normal (*p* ≤ 0.05), *n* = 10/group. Representative low- and high-magnification images of **b** iNOS and **d** Arg1 in the ventral aspect of the optic nerve are shown in control uninjured, 3, 7, and 28 days after injury. Examples of iNOS- and Arg1-positive cells are indicated by “>.” Scale bar of **b** and **d**: 40 μm top images and 10 μm bottom images for each

**Fig. 3:**
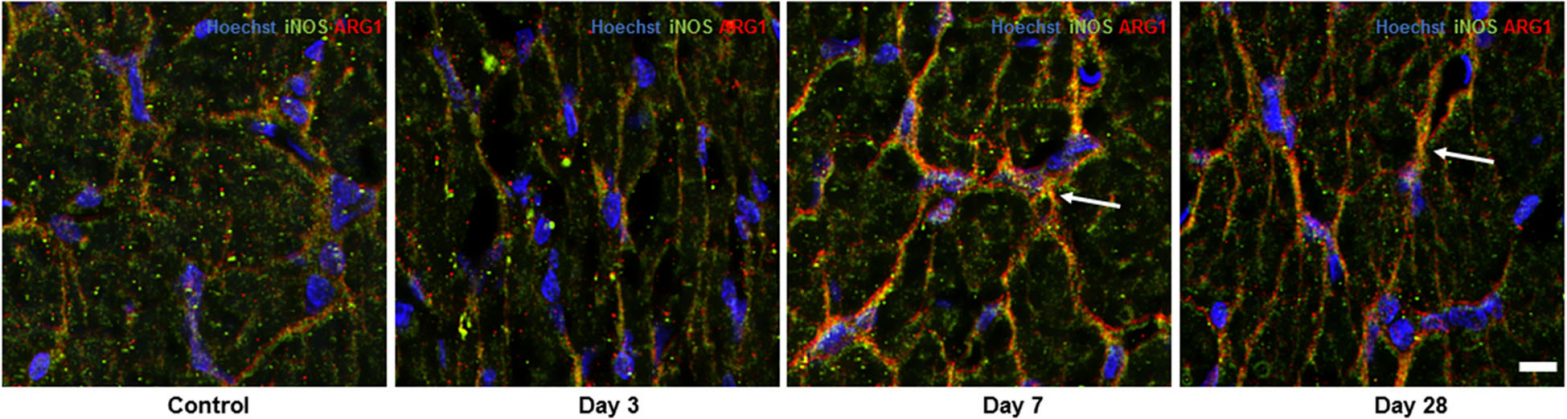
Representative images of iNOS and Arg-1 colocalization in ventral ON vulnerable to secondary degeneration. iNOS+/Arg1+cells are yellow and indicated by white arrows, particularly apparent 7 and 28 days after injury to the optic nerve, *n* = 6/group. Scale bar: 10 μm

Semi-quantitative assessment 3, 7, and 28 days after injury in the ventral portion of the right optic nerve directly below the dorsal primary injury site revealed significant increases in the immunointensity of both the pro-inflammatory biomarker inducible nitric oxide synthase (iNOS) (Fig. 2a, b) and anti-inflammatory biomarker arginase-1 (Arg-1) (Fig. 2c, d) at 7 days (*p* ≤ 0.05, *F* = 5.04, df = 38 and *p* ≤ 0.05, *F* = 7.41, df = 38, respectively). Immunointensity of iNOS returned to control levels 28 days after injury (*p* > 0.05), while arg-1 remained elevated (*p* ≤ 0.05). Contrary to the microglia/macrophage “polarization” phenotype previously described [40], we demonstrate colocalization of iNOS and Arg1 immunoreactivity within individual cells, evident 7 and 28 days after injury (Fig. 3).

**Fig. 4:**
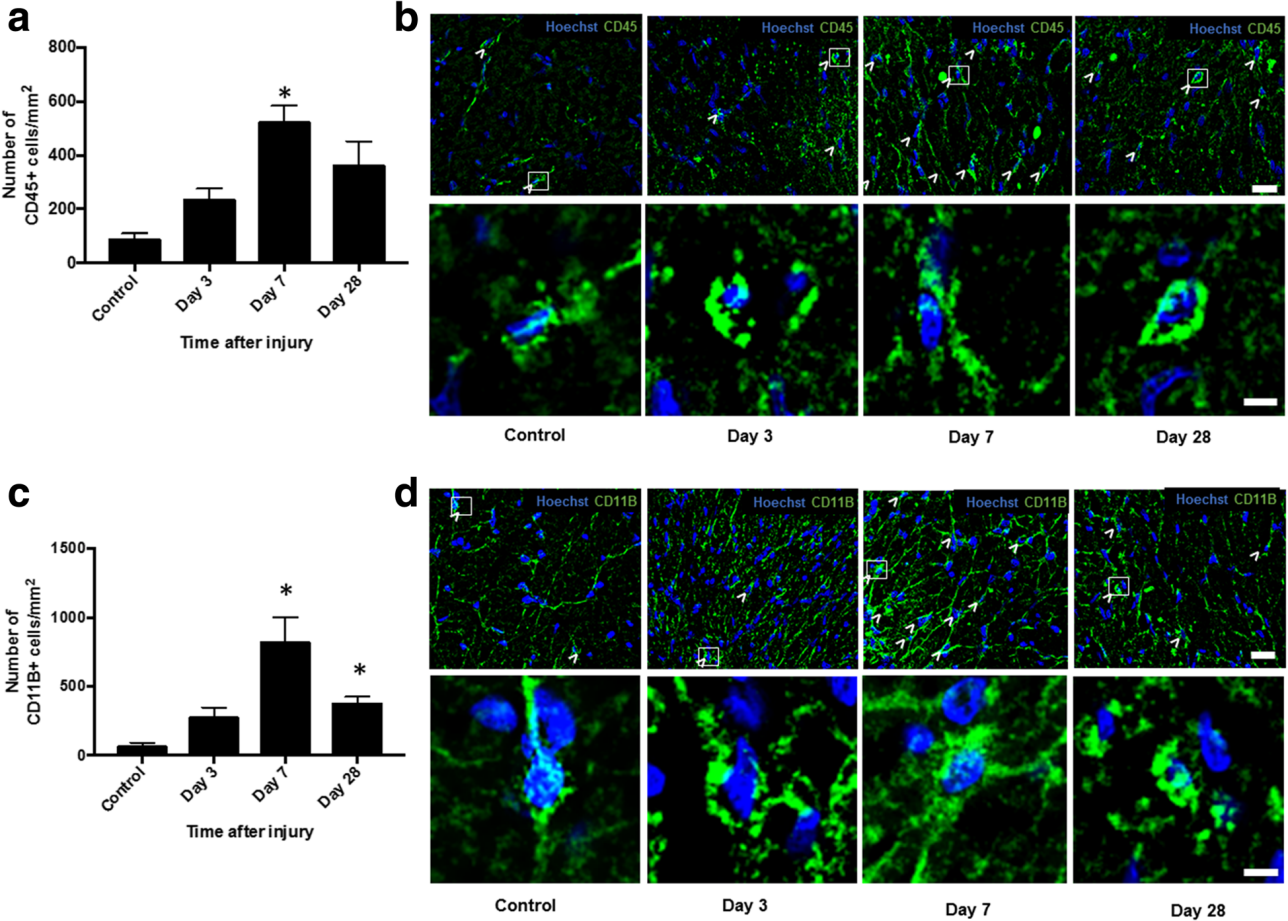
Quantification of CD11B+ and CD45+ cells in ventral ON vulnerable to secondary degeneration. Mean ± SEM numbers of **a** CD45+ and **c** CD11b+ cells in control, uninjured optic nerve and at 3, 7, and 28 days following partial optic nerve transection, in ventral optic nerve vulnerable to secondary degeneration are shown. * indicates significantly different from normal (*p* ≤ 0.05), *n* = 6/group. Representative low- and high-magnification images of **b** CD45 and **d** CD11b in the ventral aspect of the optic nerve are shown in control uninjured, 3, 7, and 28 days after injury. Examples of CD45+ and CD11b+ cells, boxed cells at low magnification, are shown in high magnification. Scale bar of **b** and **d**: 20 μm top images and 3 μm bottom images for each

The numbers of cells expressing CD11b (complement 3 receptor) a constitutive marker of leukocytes and CD45 which is expressed on all nucleated hematopoietic cells was assessed in the ventral portion of the right optic nerve. Quantification of CD45+ and CD11b+ microglia and macrophages demonstrated significant increases in the numbers of both CD45+ and CD11b+ cells 7 days after injury (*p* ≤ 0.05, *F* = 4.74, df = 23 and *p* ≤ 0.05, *F* = 9.15, df = 23, respectively) (Fig. 4a, c). The numbers of CD11b+ cells remained significantly higher than in uninjured controls at 28 days post-injury (*p* ≤ 0.05) (Fig. 4c, d), whereas CD45+ cells returned to levels not different from control at this time point (*p* > 0.05) (Fig. 4a, b).

### Inflammatory cell responses in the brain

**Fig. 5:**
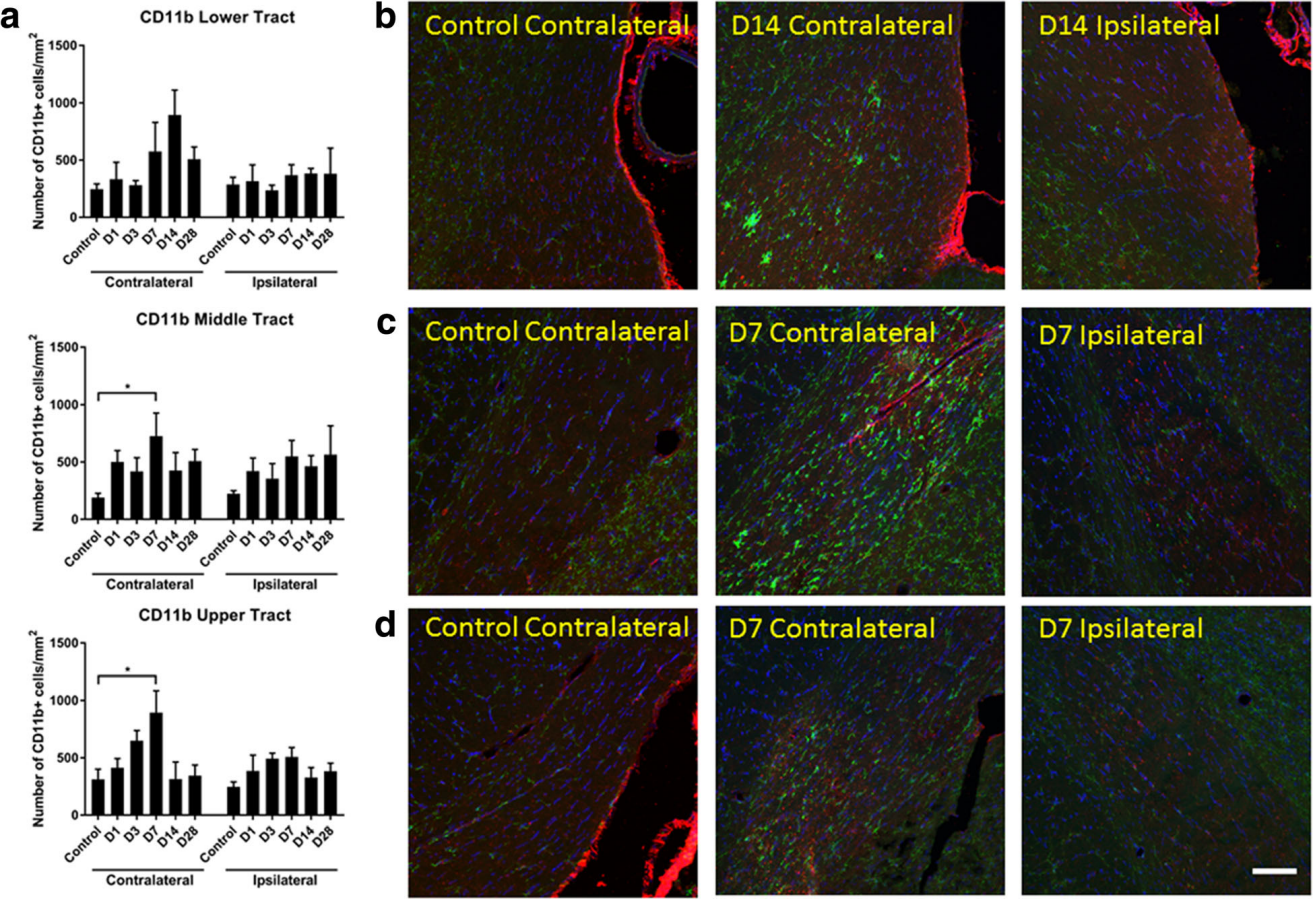
Quantification of CD11b+ cells in the optic tract of the brain. Mean ± SEM numbers of **a** CD11b+ cells in the lower, middle, and upper regions of the optic tract in control uninjured optic nerve, compared to 1, 3, 7, 14, and 28 days following partial optic nerve transection, assessed separately for the right and left hand sides of the brain, *n* = 5–6/group. *p ≤ 0.05, **p ≤ 0.01, ***p ≤ 0.001, ****p ≤ 0.0001. Representative images of infiltrating CD11b+ cells in the **b** lower, **c** middle, and **d** upper optic tract, in control uninjured, and 7–14 days following partial optic nerve transection: D is day. Scale bar of **b**–**d**: 100 μm

Changes in densities of inflammatory cell populations were assessed in the lower, middle, and upper optic tracts (i.e., progressing from juxta-chiasmal = lower; mid-way between the chiasm and the superior colliculus = middle; pre-superior colliculus = upper) of the brain at 1, 3, 7, 14, and 28 days following partial injury to the optic nerve. Time points were chosen to complement the optic nerve analyses of the present study together with published work assessing microglia and macrophages in the partial optic nerve cut model [25, 39]. CD11b+ cells were assessed on both the left and right optic tracts, corresponding to the contralateral and ipsilateral sides to the injury, respectively. Two-way ANOVA revealed no significant changes in the densities of CD11b+ leukocytes in the lower tracts, compared to uninjured controls (*p* = 0.17, *F* = 1.63, df = 5) (Fig. 5a). There were changes in the number of CD11b+ cells/mm^2^ in the middle (two-way ANOVA: *p* = 2 × 10^−3^, *F* = 4.53, df = 5; contralateral: *p* = 0.02, *F* = 3.09, df = 5; ipsilateral: *p* = 0.17, *F* = 1.69, df = 5) and upper tracts (two-way ANOVA: *p* = 1 × 10^−4^, *F* = 6.217, df = 5; contralateral: *p* = 9 × 10^−3^, *F* = 3.95, df = 5; ipsilateral: *p* = 0.051, *F* = 2.561, df = 5), with post hoc analysis revealing a significant increase relative to uninjured controls 7 days post-injury on the contralateral side (*p* = 0.03, middle tract; *p* = 0.01, upper tract), returning to control levels at day 14 (Fig. 5b, c).

**Fig. 6:**
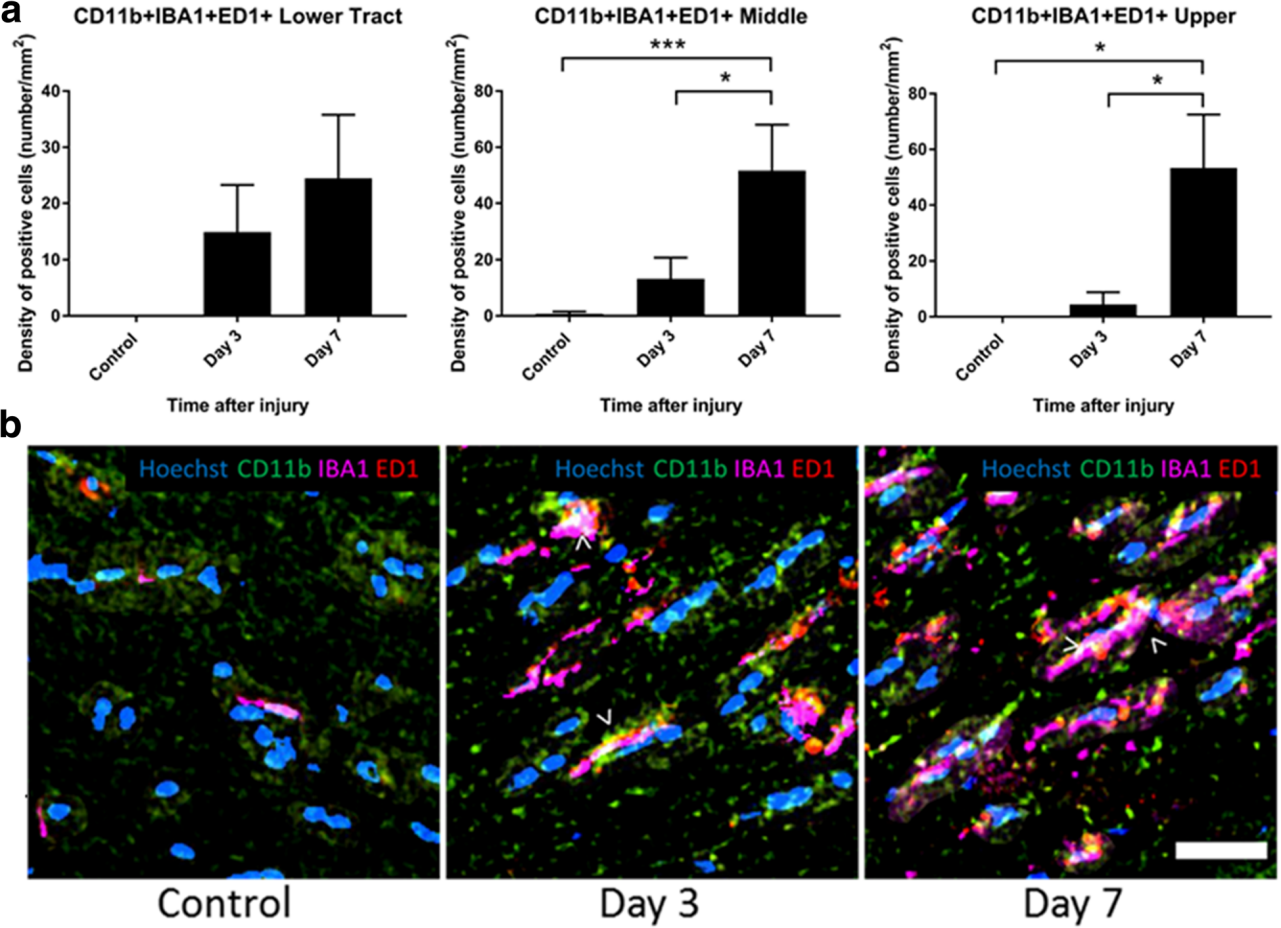
Quantification of CD11b+/IBA1+/ED1+ cells in the optic tract of the brain. Mean ± SEM numbers of **a** CD11b+/IBA1+/ED1+ cells across the lower, middle, and upper regions of the optic tract in control uninjured optic nerve, compared to 3 and 7 days following partial optic nerve transection. *n* = 6 for control, *n* = 5 for 3 and for 7 days. *p ≤ 0.05, **p ≤ 0.01, ***p ≤ 0.001, ****p ≤ 0.0001. Representative images of **b** CD11b+/IBA1+/ED1+ cells, indicated by “>,” in the lower region of the optic tract in control uninjured, 3 and 7 days following partial optic nerve transection. Scale bar of **b**: 5 μm

**Fig. 7:**
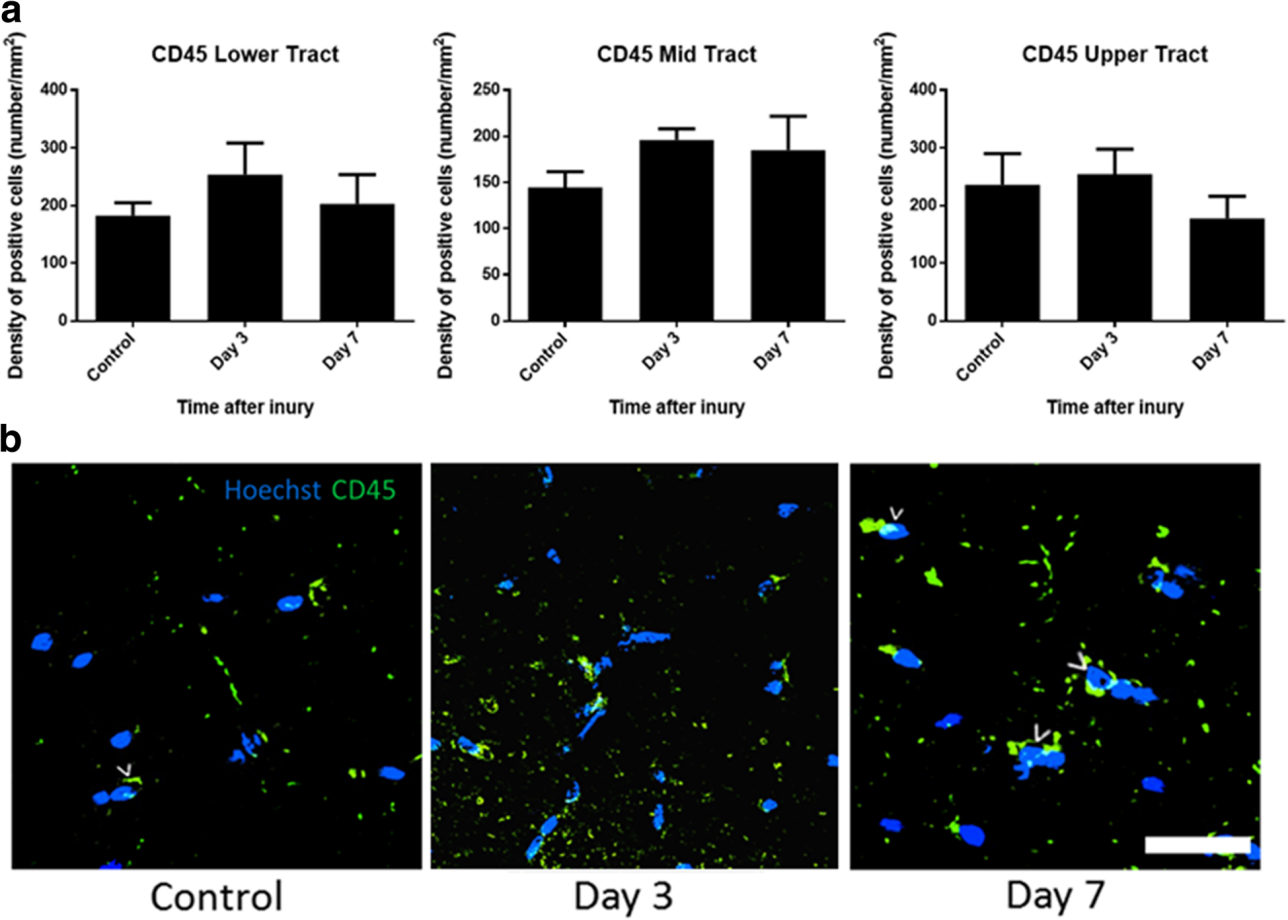
Quantification of CD45+ cells in the optic tract of the brain. Mean ± SEM numbers of CD45+ cells **a** across the lower, middle, and upper regions of the optic tract in control uninjured optic nerve, compared to 3 and 7 days following partial optic nerve transection. *n* = 6 for control, *n* = 5 for 3/7 days. Representative images of CD45+ cells (**b**), indicated by “>” in the lower region of the optic tract in control uninjured optic nerve, and 3 and 7 days following partial ON transection. Scale bar for **b**: 3 μm

Additional analyses were conducted focusing on the contralateral optic tract, to investigate first increases in further microglial/ macrophage subpopulations in a subset of sections of the optic tract at 1, 3, and 7 days following partial injury to the optic nerve. CD11b+ cells that also colocalized with IBA1 (a microglia/ macrophage marker which recognizes both resident and activated microglia/ macrophages) and ED1 (a marker of resident and infiltrating activated macrophages) were unchanged in the lower tract at the measured time points (*p* = 0.14, *F* = 2.29, df = 2) but significantly increased at 7 days compared to uninjured controls in the middle (*p* = 6 × 10^−4^, *F* = 13.54, df = 2) and upper tract (*p* = 0.03, *F* = 20.33, df = 2) (Fig. 6a, b), indicating that activation was enhanced in these regions. In contrast to outcomes within the optic nerve, there were no changes in the density of CD45^+^-positive cells in the contralateral optic tract. Quantification of CD45+ microglia and macrophages did not reveal any significant changes at 3 or 7 days following injury in any of the optic tract regions analyzed (*p* = 0.57, *F* = 0.59, df = 2 for lower; *p* = 0.41, *F* = 0.96, df = 2 for middle; and *p* = 0.60, *F* = 0.53, df = 2 for upper optic tract) (Fig. 7a, b).

**Fig. 8:**
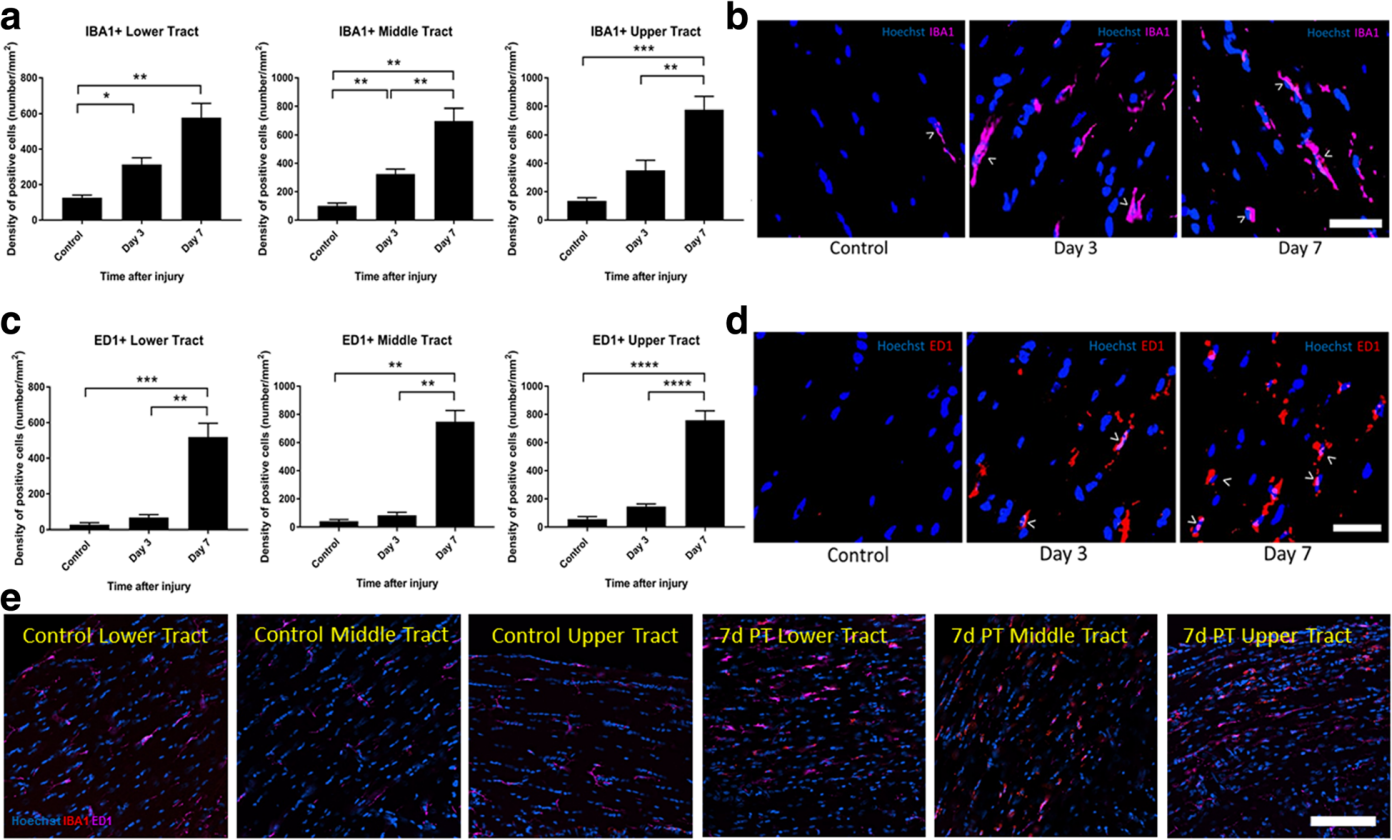
Quantification of IBA1+ and ED1+ cells in the optic tract of the brain. Mean ± SEM numbers of **a** IBA1+ and **c** ED1+ cells across the lower, middle, and upper regions of the optic tract in control uninjured optic nerve, compared to 3 and 7 days following partial optic nerve transection. *n* = 6 for control, *n* = 5 for 3 and for 7 days. *p ≤ 0.05, **p ≤ 0.01, ***p ≤ 0.001, ****p ≤ 0.0001. Representative images of **b** IBA1+ and **d** ED1+ cells at high magnification, indicated by “>,” in the lower region of the optic tract in control uninjured optic nerve, and 3 and 7 days following partial optic nerve transection. Scale bar for **b**–**d**: 5 μm. **e** Representative images of IBA1+ and ED1+ cells at low magnification, in the lower middle and upper regions of the optic tract in control uninjured optic nerve, and 7 days following partial optic nerve transection. Scale bar: 100 μm

Cells positive for the markers IBA1 and ED1 in isolation were also quantified in the contralateral optic tract at 3 and 7 days post-injury. Density of IBA1+ cells significantly increased from uninjured controls at 3 days post-injury, with a further increase at 7 days post-injury in the lower (*p* = 1 × 10^−4^, *F* = 20.08, df = 2; 3 days *p* = 0.01, 7 days *p* = 0.01) and middle (*p* = 7 × 10^−6^, *F* = 33.48, df = 2; 3 days *p* = 3 × 10^−3^, 7 days *p* = 4 × 10^−3^) regions of the optic tract. Increases in the upper tract only reached significance at 7 days (*p* = 3 × 10^−5^, *F* = 28.38, df = 2; 7 days *p* = 2 × 10^−5^) (Fig. 8a, b, e; high and low magnification representative images, respectively). The densities of ED1+ cells increased at 7 days following injury in the lower, middle, and upper tracts (*p* = 7 × 10^−5^, *F* = 23.33, df = 2; *p* = 0.00, *F* = 77.39, df = 2 ;and *p* = 0.00, *F* = 87.61, df = 2, respectively; 7 days *p* = 6 × 10^−3^, *p* = 1 × 10^−4^, *p* = 0.00, respectively) (Fig. 8c, d, e; high and low magnification representative images, respectively).

**Fig. 9:**
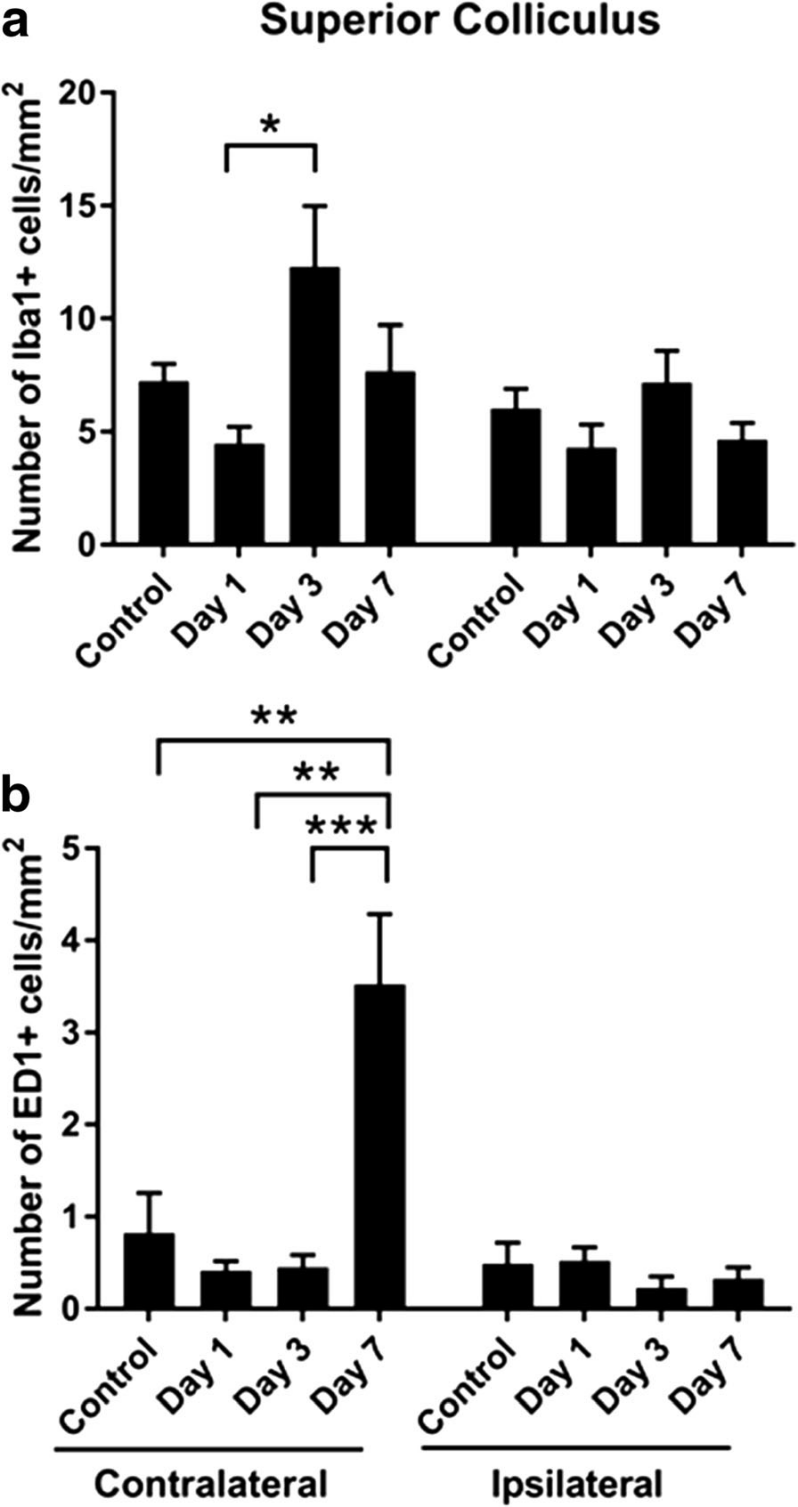
Quantification of IBA1+ and ED1+ cells in the superior colliculus. Mean ± SEM numbers of **a** IBA1+ and **b** ED1+ cells across the lower, middle, and upper regions of the optic tract in control uninjured optic nerve, compared to 3 and 7 days following partial optic nerve transection. *n* = 6 for control, *n* = 5 for 3 and for 7 days. *p ≤ 0.05, **p ≤ 0.01, ***p ≤ 0.001, ****p ≤ 0.0001

IBA1+ and ED1+ cells were also quantified in the superior colliculus at 1, 3, and 7 days post-injury, assessing both the contralateral and ipsilateral sides. IBA1+ cells did not significantly change relative to control uninjured nerve on either the contralateral or ipsilateral sides (*p* = 0.057, *F* = 4.21, df = 3; ipsilateral: *p* = 0.25, *F* = 1.46, df = 3) (Fig. 9a). However, post hoc tests revealed that ED1+ cells were significantly increased at day 7 on the contralateral side (*p* = 0.002) (contralateral: *p* = 2 × 10^−4^, *F* = 10.47, df = 3; ipsilateral: *p* = 0.50, *F* = 0.80, df = 3) (Fig. 9b).

### Blood-brain barrier integrity

**Fig. 10:**
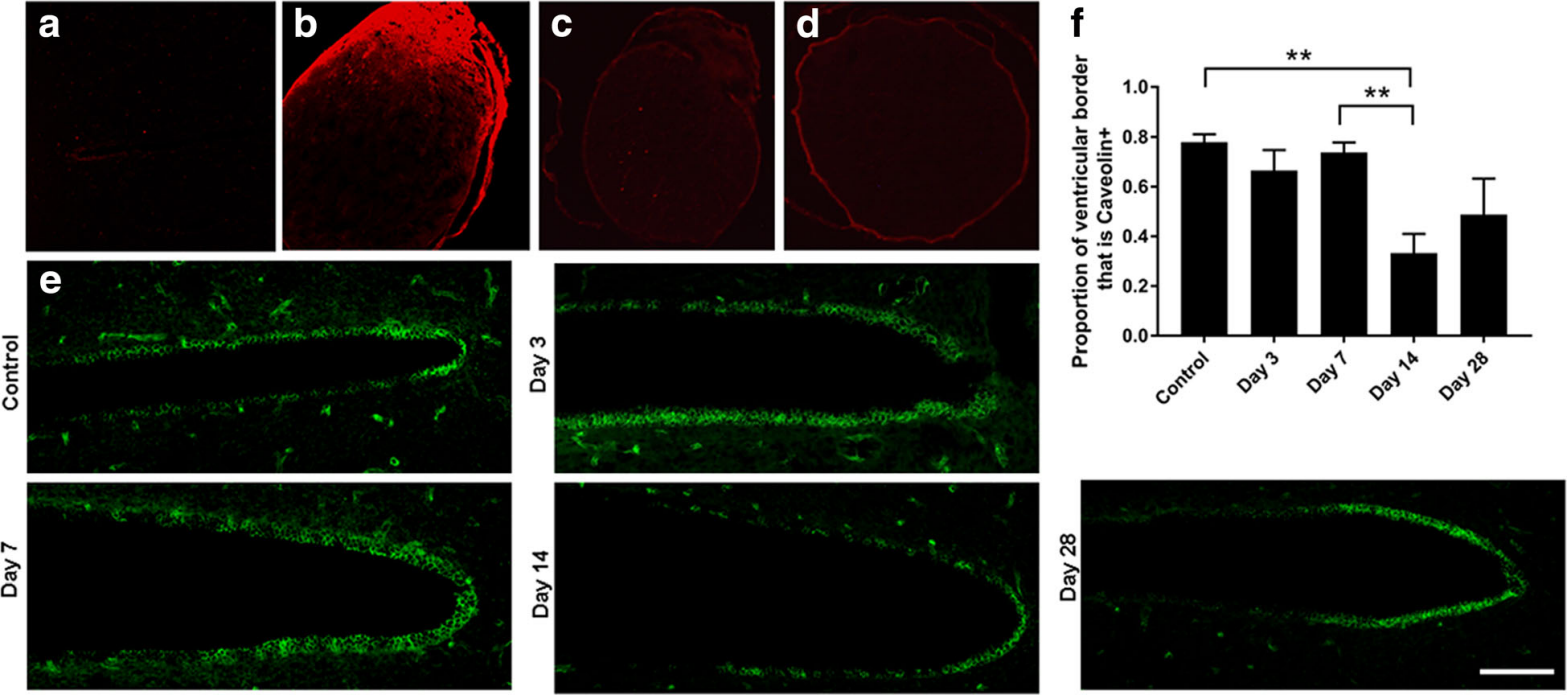
Evans blue fluorescence and caveolin immunoreactivity in the brain following partial optic nerve transection. Lack of Evans blue fluorescence in a representative image of the periventricular region of the brain at 3 days after partial optic nerve transection (**a**), compared to strongly positive fluorescence at the partial optic nerve injury site 7 days after injury (**b**). Evans blue fluorescence in optic nerve 28 days after injury (**c**) was not different from uninjured controls injected with Evans blue (**d**). Caveolin immunoreactivity in endothelial cells lining the ventricles at 3, 7, 14, and 28 days after injury (**e**), scale = 100 μm. Mean ± SEM proportion of the ventricle lined with caveolin-1+ cells (**f**). *n* = 7–12. *p ≤ 0.05, **p ≤ 0.01, ***p ≤ 0.001, ****p ≤ 0.0001

Extravasation of Evans blue dye into the brains of rats following partial optic nerve injury was assessed qualitatively, being mindful of the caveats of this technique for assessing BBB integrity [41]. Despite our earlier observations of Evans blue dye in the brain at days 1 and 3 following this injury using multispectral imaging and quantitative assessment of extravasated Evans blue respectively [38], we did not observe Evans blue fluorescence at above background levels in cryosections of the brain viewed by confocal microscopy in the current study (Fig. 10a). The lack of Evans blue fluorescence in the brain sections in the current study may have been due to storage in sucrose and PBS prior to sectioning, and the single washing of the brain sections required to remove the OCT prior to section mounting. Previously, we observed Evans blue dye in the optic nerve at days 1 and 3 following injury [38]. In the current study, assessment of later time points indicated the presence of intense Evans blue staining at the optic nerve injury site and low-intensity diffuse staining ventral to the site of injury at 7 days post-injury (Fig. 10b). However, Evans blue fluorescence was not different to control at 28 days after partial transection (Fig. 10c, d), indicating closure of the BBB in the optic nerve at this time point after injury.

Caveolin-1 is a structural component of caveolae, mediates transcytosis through endothelial cells via interactions with claudin [42], and can be used as an indicator of BBB integrity [38]. Caveolin-1 immunoreactivity surrounding the third ventricle was assessed at 3, 7, 14, and 28 days following injury, to provide insight regarding the time of closure of the BBB following remote injury. The percentage of the third ventricle lined with caveolin-1+ cells was not different to control at 3, 7, and 28 days following partial optic nerve transection but was significantly reduced at 14 days (ANOVA: *p* = 5 × 10^−4^, *F* = 6.52, df = 4; 14 days *p* = 5 × 10^−3^) (Fig. 10e, f), indicating a transient compromise of caveolin-1-mediated junctions at this later time point.

**Fig. 11:**
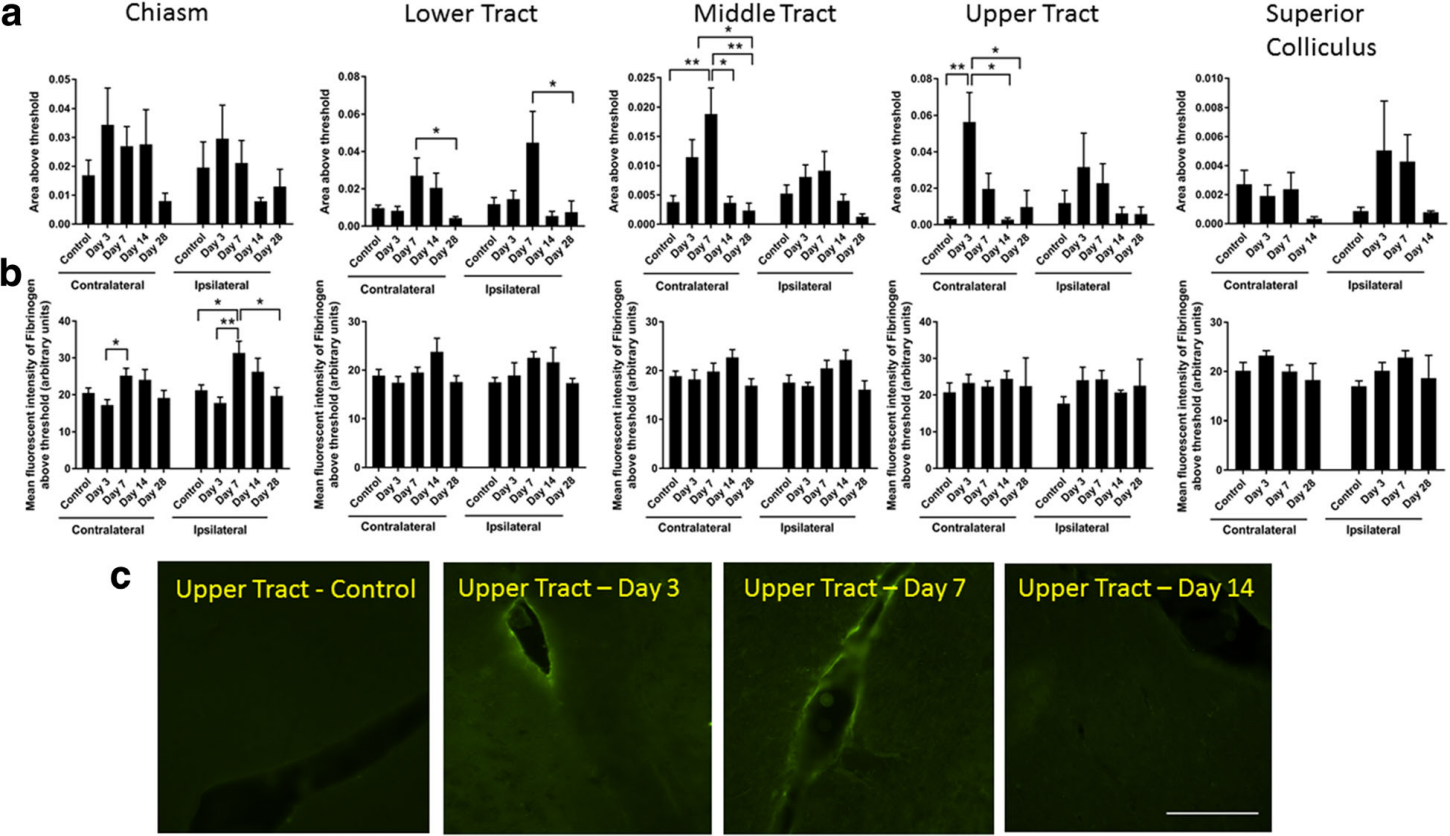
Fibrinogen immunoreactivity in the brain following partial optic nerve transection. Mean ± SEM fibrinogen immunoreactivity assessing **a** area and **b** intensity above a constant set threshold in control uninjured optic nerve, compared to 1, 3, 7, 14, and 28 days, assessed separately for the right- and left-hand sides of the brain. Lower, middle, and upper regions of the optic tract were assessed separately. *n* = 5–6/group. *p ≤ 0.05, **p ≤ 0.01, ***p ≤ 0.001, ****p ≤ 0.0001. Representative images of the upper tract (**c**) show breaches in the BBB indicated by increased areas of diffuse fibrinogen immunoreactivity (bracketed), 3 days after injury, compared to control uninjured, and 7 and 14 days following partial optic nerve transection. Scale bar for **c**: 100 μm

Given the potential for washout of Evans blue dye from the tissue sections, immunoreactivity of fibrinogen was used as an additional measure of BBB integrity. Fibrinogen is a blood-derived protein whose accumulation in the brain indicates loss of BBB integrity [43]. The area of fibrinogen immunoreactivity, indicative of the size of the breach of the BBB, did not change with injury in the optic chiasm (two-way ANOVA: *p* = 0.24, *F* = 1.41, df = 4) (Fig. 11a). However, the intensity of staining in the chiasm, perhaps indicating a focal breach, did change with injury (contralateral: *p* = 0.03, *F* = 3.10, df = 4; ipsilateral: *p* = 2 × 10^−3^, *F* = 5.18, df = 4), with post hoc analysis revealing that increases in signal intensity relative to control were confined to the ipsilateral side at 7 days post-injury (*p* = 0.03) (Fig. 11b). Changes in the area of fibrinogen immunoreactivity in the lower tract were seen on both sides (contralateral: *p* = 0.02, *F* = 3.33, df = 4; ipsilateral: *p* = 0.03, *F* = 2.98, df = 4); however, post hoc tests only revealed differences between 7 and 28 days, rather than relative to control (contralateral: *p* = 0.03; ipsilateral: *p* = 0.04) (Fig. 11a). No differences in the intensity of fibrinogen immunoreactivity were observed in the lower tract (contralateral: *p* = 0.10, *F* = 2.09, df = 4; ipsilateral: *p* = 0.27, *F* = 1.35, df = 4). In the middle tract, changes in the area of fibrinogen immunoreactivity were confined to the contralateral side (contralateral: *p* = 3 × 10^−4^, *F* = 7.12, df = 4; ipsilateral: *p* = 0.06, *F* = 2.48, df = 4), with post hoc analysis revealing differences relative to control 7 days post-injury (*p* = 4 × 10^−3^). There were no significant differences in the intensity of fibrinogen immunoreactivity in the middle tract (contralateral: *p* = 0.24, *F* = 1.45, df = 4; ipsilateral: *p* = 0.06, *F* = 2.55, df = 4). Changes in the upper tract were similar to the middle tract but occurred earlier (contralateral: *p* = 0.001, *F* = 5.98, df = 4; ipsilateral: *p* = 0.69, *F* = 0.56, df = 4) with post hoc tests showing that area of the breach increased at day 3 on the contralateral side (*p* = 0.004) (Fig. 11a, c). No changes were observed in the intensity of fibrinogen immunoreactivity in the upper tract (contralateral: *p* = 0.086, *F* = 2.26, df = 4; ipsilateral: *p* = 0.10, *F* = 2.10, df = 4) (Fig. 11b). Finally, in the superior colliculus, no significant changes in fibrinogen immunoreactivity were observed for area (contralateral: *p* = 0.058, *F* = 2.95, df = 3; ipsilateral: *p* = 0.06, *F* = 2.82, df = 3) or intensity of immunoreactivity (contralateral: *p* = 0.32, *F* = 1.23, df = 3; ipsilateral: *p* = 0.16, *F* = 1.88, df = 3) (Fig. 11a, b).

### Cytokine expression is altered in rat brains in a time-dependent manner following partial injury to the optic nerve

**Fig. 12:**
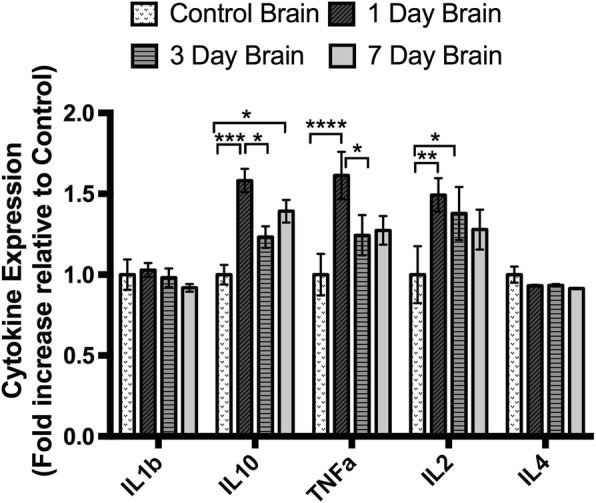
Multiplex analysis of cytokines in the brain following injury. Data are expressed as mean ± standard error of mean. *p ≤ 0.05, **p ≤ 0.01, ***p ≤ 0.001, ****p ≤ 0.0001, one-way ANOVA (Tukey’s correction)

The inflammatory response in the whole brain homogenates following partial injury to the optic nerve was determined by measuring changes in cytokine protein levels in homogenized rat brains at 1, 3, and 7 days post-injury compared to uninjured controls (Fig. 12). While it would perhaps have been preferable to assess the cytokines in the individual optic tract regions of interest, the volume of tissue required for these assays precluded these assessments. In these experiments, ten cytokines were measured: IL-6, IL-1β, TNFα, IL-2, IL-4, IL-10, IL-12, GMCSF, IL-1α, INFγ; however, differences between injured and control rat brains were only detected for three of them, TNFα, IL-2, and IL-10. The pro-inflammatory cytokine, IL-1β, and the anti-inflammatory cytokine, IL-4, remained unaltered in injured rat brains compared to controls, and five cytokines were below the detection limit of the multiplex assay, IL-6, IL-12, GMCSF, IL-1α, and INFγ. Tumor necrosis factor (TNFα) is a multifunctional cytokine most often referred to as a potent pro-inflammatory cytokine, produced by microglia and astrocytes. There was a significant increase in TNFα protein levels in rat brains in the early time point, 1 day post-injury (1.6-fold increase ± 0.14) compared to uninjured controls (*p* < 0.0001, *F* = 4.13, df = 3) (Fig. 12). At 3 days post-injury, the concentration of TNFα returned to levels that were not significantly different from uninjured controls (*p* > 0.05), and these levels were maintained at 7 days post-injury (Fig. 12). The pro-inflammatory cytokine IL-2 was significantly elevated in the rat brains at an early time point following injury, 1 day (1.4-fold increase, *p* < 0.05, *F* = 2.12, df = 3), remained elevated at 3 days post-injury (1.4-fold increase, *p* < 0.05, *F* = 2.12, df = 3), and decreased to levels not significantly different from controls at 7 days post-injury (Fig. 12). IL-10 is regarded primarily as an anti-inflammatory cytokine, having a potent inhibitory effect on the production of several pro-inflammatory mediators including TNFα and IL-1β. IL-10 levels were biphasic, being significantly elevated from control levels at both an early time point, 1 day (1.6-fold increase, *p* < 0.001, *F* = 13.28, df = 3), and a later time point, 7 days (1.4-fold increase, *p* < 0.05, *F* = 13.28, df = 3) post-injury, with a decline to control levels in between at 3 days post-injury (Fig. 12).

## Discussion

**Fig. 13:**
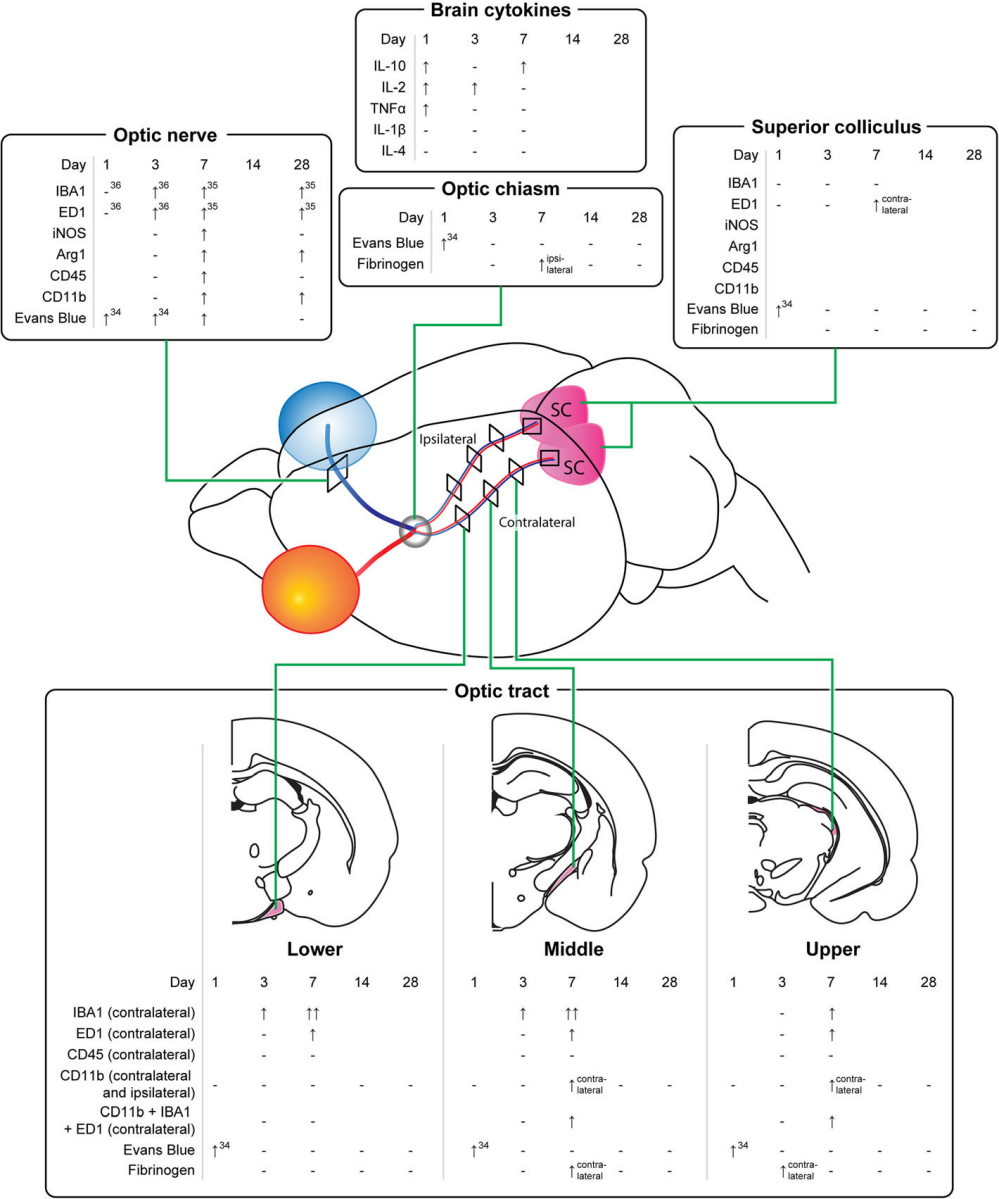
Summary of study outcomes. ↑ indicates a significant increase relative to uninjured control, ↑↑ indicates a further increase, and ↓ indicates a significant decrease (*p* ≤ 0.05) in a measured marker relative to control; “-” indicates no change relative to control. A space indicates not yet tested. Note that data for the optic tract are referring to the contralateral left-hand side, unless specifically indicated. Small numbers refer to references to published literature

The current study has provided evidence that an injury to the optic nerve instigates a remote response in the brain in a time-dependent manner, with production of both pro- and anti-inflammatory cytokines and increases in resident and infiltrating inflammatory cells (for summary of outcomes including relevant published data in this model, see Fig. 13). Cytokines were rapidly increased in the brain, prior to changes in the measured inflammatory cell populations in the ventral portion of the optic nerve, immediately adjacent to the injury. Inflammatory cell infiltration was evident in well-defined regions of the brain with significant accumulation being limited to the contralateral side of the brain. Specific populations of inflammatory cells were increased in a region-specific manner. Increases in the density of resident IBA1+ cells were seen in the lower and middle regions of the contralateral optic tract and the superior colliculus as early as 3 days post-injury. However, increases in the number of leukocytes and activated microglia and macrophages relative to control, indicated by CD11b+ and ED1+ cells, were only evident 7 days post-injury in the upper tract regions and in the contralateral side of the superior colliculus, indicating progression of damage both towards and from the synaptic terminals. This progression of activated inflammatory cells was further confirmed by an increase in triple-positive CD11b/IBA1/ED1 cells in both the middle and upper regions of the contralateral optic tract at 7 days post-injury. Increases in microglia and macrophages in the ipsilateral optic tract were minimal and not significant. Importantly, breaches to the BBB can precede inflammation, particularly apparent in the upper tract in this study. Taken together, the data indicate that an injury to the optic nerve results in rapid changes towards the synaptic terminals of affected retinal ganglion cells, which initiate localized inflammatory cell infiltration perhaps potentiated by early increases in selected cytokines. Further studies will be required to determine whether similar changes also occur in an anterograde trans-synaptic fashion, for example, in cortically connected regions [44, 45].

Delayed secondary damage often occurs to remote brain regions that are anatomically connected to the primary infarct by axonal projections. Studies in rats using middle cerebral artery occlusion as an ischemia model consistently show that distinct regions of the thalamus with axonal connections to the primary infarct develop gliosis, inflammation, and significant neuronal loss at delayed post-stroke time points [29]. In our current study, a commonality among all observations of altered inflammatory cell density and BBB permeability in the brain is the axonal connectedness of these regions with the primary injury site, i.e., most significant alterations are observed in the contralateral optic tract and superior colliculus, which house the majority of injured axons that cross over at the optic chiasm, suggesting a role for axonal degeneration and potential white matter pathology, suggested to be of functional relevance for post-stroke recovery [33]. Our findings indicate that remote damage is limited to areas connected to the primary injury site. However, it is important to note some limitations of the current study. Only one section per brain region was analyzed, and we did not examine all visual projections, such as the lateral geniculate nucleus. In addition, the analyses of cytokines used whole brain homogenates and thus were not confined to axonally connected pathways. Nevertheless, we observed a significant increase in the anti-inflammatory cytokine, IL-10, at 7 days post-injury, which coincided with increased numbers of microglia in the brain, perhaps indicating a concerted response by anti-inflammatory cell subtypes at this time. Depletion of CD11b+ cells worsens damage 24 h following ischemic injury, associated with increased pro-inflammatory indicators, and similar mechanisms may act here [46]. Further, more focused assessments may provide additional insight. It is also important to note that PVG rats have particular susceptibilities to immune challenges [47]. Indeed, there are strain-specific differences for many outcomes in rats [48]. PVG rats were initially chosen for our studies using the partial optic nerve transection model of secondary degeneration, as they are not albino and therefore have better vision for assessments of visual function following injury. Female rats were chosen for ease of housing and to provide much needed and oft-missing information on injury outcomes in female animals.

Using Evans blue dye, we have observed breaches in the BBB in the brain as early as 1 day after this partial optic nerve injury [38], at the same time as both the current observed increases in both pro- (IL-2, TNFα) and anti- (IL-10) inflammatory cytokines in whole brain homogenates. While IL-2 and TNFα can cross the BBB [49, 50], and IL-10 cannot [51] the breached BBB may have facilitated entry of cytokines, elevated as a result of optic nerve injury, into the brain from the circulation. The delayed increases in inflammatory cell numbers observed in the middle contralateral optic tract at 7 days post-injury coincided with a significant increase in the area of fibrinogen-positive signal in this brain region, indicating associated alterations in BBB permeability. Increases in microglia- and macrophage-derived cytokines including TNFα correlate with immune cell infiltration and have been implicated in the modulation of endothelial cell function [28] and changes in BBB permeability [52]. Reactive oxygen species generated by macrophages [7] also compromise BBB integrity [50]. Thus, while BBB breaches in the upper tract precede, and may contribute to increases in inflammatory cells, multiple factors may be contributing to a feed forward system exacerbating the damage. While it is clear from our earlier published work that infiltration of microglia and macrophages also occurs at the partial optic nerve injury site and spreads in an efferent direction towards the brain along the nerve vulnerable to secondary degeneration [38, 39], it now becomes apparent that as time passes, inflammation may also move from degeneration in the upper tract and synaptic terminals back towards the injury. The anti-inflammatory cytokine IL-10 increases in brain homogenates in a biphasic pattern, being elevated at 1 day, returning to control levels at 3 days and elevated at 7 days after injury, likely as a compensatory response, associated with the later changes in microglia/ macrophage activation and phagocytosis. Further studies assessing cytokines in specific brain regions identified in this study as sites of inflammatory cell infiltration, pooling tissue from multiple animals, will be required to determine the direct relationships between cytokine changes, cellular infiltrations, and BBB breach. At later times after injury, there is a disruption to caveolin-1 immunoreactivity lining the ventricles, indicating that compromise of the BBB is not swiftly resolved and that injury to the optic nerve has persisting remote consequences in the brain.

While the increase in CD45+ cells in the optic nerve was transient, decreasing to control levels at 28 days following injury, the number of CD11b+ cells in the ventral optic nerve remained elevated at 28 days following injury. Similarly, iNOS and Arg1 expression were significantly increased within the ventral optic nerve 7 days after injury compared to uninjured controls, with only iNOS levels decreasing to control at 28 days post-injury. Interestingly, cells showed concurrent expression of iNOS and Arg1 indicating that these immunoreactive cells exhibit a mixed phenotype, as previously shown following traumatic brain injury [18]. Similar findings have also been reported in the cortex following spared nerve injury to the sciatic nerve [26]. As both microglia and infiltrating non-myeloid-derived leukocytes express CD11b and CD45 markers, but differ in the levels of expression of these markers, i.e., infiltrating leukocytes have high CD45 expression compared to resident microglia, we were unable to differentiate microglia from macrophages and leukocytes in general in this instance. Interestingly, CD45+ cells were not elevated in the optic tract, indicating differences in the features of the adjacent and remote inflammatory responses.

## Conclusion

In summary, our study demonstrates that a partial injury to the optic nerve induces transient changes in whole brain cytokine expression and increased inflammatory cell density and BBB permeability remotely along the visual pathways in the brain. Significant alterations are restricted to the contralateral side of the brain, suggesting that, at least in this traumatic injury model, inflammatory and BBB damage is confined to regions that are “axonally connected” to the primary injury site, although it remains to be determined whether such damage also extends trans-synaptically.

## Abbreviations

ANOVA: Analysis of variance
Arg1: Arginase 1
BBB: Blood-brain barrier
CNS: Central nervous system
df: Degrees of freedom
GMCSF: Granulocyte-macrophage colony-stimulating factor
IFN: Interferon
IL: Interleukin
iNOS: Inducible nitric oxide synthase
OCT: Optimal cutting temperature
TNFα: Tumor necrosis factor alpha

## References

[1] Jia Z, Zhu H, Li J, Wang X, Misra H, Li Y (2012). Oxidative stress in spinal cord injury and antioxidant-based intervention. Spinal Cord.

[2] Oyinbo CA (2011). Secondary injury mechanisms in traumatic spinal cord injury: a nugget of this multiply cascade. Acta Neurobiol Exp.

[3] David S, Kroner A (2011). Repertoire of microglial and macrophage responses after spinal cord injury. Nat Rev Neurosci.

[4] Perez-Polo JR, Rea HC, Johnson KM, Parsley MA, Unabia GC, Xu G, Infante SK, DeWitt DS, Hulsebosch CE (2013). Inflammatory consequences in a rodent model of mild traumatic brain injury. J Neurotrauma.

[5] Loane DJ, Byrnes KR (2010). Role of microglia in neurotrauma. Neurotherapeutics.

[6] Banati RB, Gehrmann J, Schubert P, Kreutzberg GW (1993). Cytotoxicity of microglia. GLIA.

[7] Doig RLOH, Bartlett CA, Maghzal GJ, Lam M, Archer M, Stocker R, Fitzgerald M (2014). Reactive species and oxidative stress in optic nerve vulnerable to secondary degeneration. Exp Neurol.

[8] Ruckh JM, Zhao J-W, Shadrach JL, PvPV W, Rao TN, Wagers AJ, RJM F (2011). Rejuvenation of regeneration in the aging central nervous system. Cell Stem Cell.

[9] Kotter MR, Li W-W, Zhao C, Franklin RJM (2006). Myelin impairs CNS remyelination by inhibiting oligodendrocyte precursor cell differentiation. J Neurosci.

[10] Kotter MR, Zhao C, Nv R, Franklin RJM (2005). Macrophage-depletion induced impairment of experimental CNS remyelination is associated with a reduced oligodendrocyte progenitor cell response and altered growth factor expression. Neurobiol Dis.

[11] Persidsky Y, Ghorpade A, Rasmussen J, Limoges J, Liu XJ, Stins M, Fiala M, Way D, Kim KS, Witte MH (1999). Microglial and astrocyte chemokines regulate monocyte migration through the blood-brain barrier in human immunodeficiency virus-1 encephalitis. Am J Pathol.

[12] Bock MD, Wang N, Decrock E, Ml B, Gadicherla AK, Culot M, Cecchelli R, Bultynck G, Leybaert L (2013). Endothelial calcium dynamics, connexin channels and blood–brain barrier function. Prog Neurobiol.

[13] Edwards JP, Zhang X, Frauwirth KA, Mosser DM (2006). Biochemical and functional characterization of three activatedmacrophage populations. J Leukoc Biol.

[14] Hsieh CL, Kim CC, Ryba BE, Niemi EC, Bando JK, Locksley RM, Liu J, Nakamura MC, Seaman WE (2013). Traumatic brain injury induces macrophage subsets in the brain. Eur J Immunol.

[15] Wang G, Zhang J, Hu X, Zhang L, Mao L, Jiang X, Liou AK-F, Leak RK, Gao Y, Chen J (2013). Microglia/macrophage polarization dynamics in white matter after traumatic brain injury. J Cereb Blood Flow Metab.

[16] Orihuela R, McPherson CA, Harry GJ (2016). Microglial M1/M2polarization and metabolic states. Br J Pharmacol.

[17] Turtzo LC, Lescher J, Janes L, Dean DD, Budde MD, Frank JA (2014). Macrophagic and microglial responses after focal traumatic brain injury in the female rat. J Neuroinflammation.

[18] Morganti JM, Riparip L-K, Rosi S (2016). Call off the dog(ma): M1/M2 polarization is concurrent following traumatic brain injury. PLoS One.

[19] Pettersen JS, Fuentes-Duculan J, Suárez-Fariñas M, Pierson KC, Pitts-Kiefer A, Fan L, Belkin DA, Wang CQF, Bhuvanendran S, Johnson-Huang LM (2011). Tumor-associated macrophages in the cutaneous SCC microenvironment are heterogeneously activated. J Invest Dermatol.

[20] Vogel DY, Vereyken EJ, Glim JE, Heijnen PD, Moeton M, Pvd V, Amor S, Teunissen CE, Jv H, Dijkstra CD (2013). Macrophages in inflammatory multiple sclerosis lesions have an intermediate activation status. J Neuroinflammation.

[21] Kim CC, Nakamura MC, Hsieh CL (2016). Brain trauma elicits non-canonical macrophage activation states. J Neuroinflammation.

[22] Ransohoff RM (2016). A polarizing question: do M1 and M2 microglia exist?. Nat Neurosci.

[23] Martinez FO, Gordon S (2014). The M1 and M2 paradigm of macrophage activation: time for reassessment. F1000Prime Reports.

[24] Perego C, Fumagalli S, Simoni M-GD (2011). Temporal pattern of expression and colocalization of microglia/macrophage phenotype markers following brain ischemic injury in mice. J Neuroinflammation.

[25] Fitzgerald M, Bartlett CA, Evill L, Rodger J, Harvey AR, Dunlop SA (2009). Secondary degeneration of the optic nerve following partial transection: the benefits of lomerizine. Exp Neurol.

[26] Xu N, Tang X-H, Pan W, Xie Z-M, Zhang G-F, Ji M-H, Yang J-J, Zhou M-T, Zhou Z-Q. Spared nerve injury increases the expression of microglia M1 markers in the prefrontal cortex of rats and provokes depression-like behaviors. Front Neurosci. 2017;11. Article 209. 10.3389/fnins.2017.00209 PMC5394168 28458629

[27] Evangelho K, Mogilevskaya M, Losada-Barragan M, Vargas-Sanchez JK. Pathophysiology of primary open-angle glaucoma from a neuroinflammatory and neurotoxicity perspective: a review of the literature. Int Ophthalmol. 2017. 10.1007/s10792-017-0795-9. 10.1007/s10792-017-0795-9 29290065

[28] Daneman R, Prat A (2015). The blood-brain barrier. Cold Spring Harb Perspect Biol.

[29] Block F, Dihné M, Loos M (2005). Inflammation in areas of remote changes following focal brain lesion. Prog Neurobiol.

[30] Weishaupt N, Zhang A, Deziel RA, AndrewTasker R, Whitehead SN (2016). Prefrontal ischemia in the rat leads to secondary damage and inflammation in remote gray and white matter regions. Front Neurosci.

[31] Viscomi MT, Molinari M (2014). Remote neurodegeneration: multiple actors for one play. Mol Neurobiol.

[32] Demeurisse G, Capon A, Verhas M, Attig E (1990). Pathogenesis of aphasia in deep-seated lesions: likely role of cortical diaschisis. Eur Neurol.

[33] Thiel A, Radlinska BA, Paquette C, Sidel M, Soucy J-P, Schirrmacher R, Minuk J (2010). The temporal dynamics of poststroke neuroinflammation: a longitudinal diffusion tensor imaging–guided PET study with 11C-PK11195 in acute subcortical stroke. The Journal of Nuclear Medicine.

[34] Finger S, Koehler PJ, Jagella C (2004). The Monakow concept of diaschisis: origins and perspectives. Arch Neurol.

[35] Hausmann ON (2003). Post-traumatic inflammation following spinal cord injury. Spinal Cord.

[36] Mietto BS, Mostacada K, Martinez AMB. Neurotrauma and inflammation: CNS and PNS responses. Mediat Inflamm. 2015;2015. article 251204. 10.1155/2015/251204 PMC4397002 25918475

[37] Echeverry S, Shi XQ, Rivest S, Zhang J (2011). Peripheral nerve injury alters blood–spinal cord barrier functional and molecular integrity through a selective inflammatory pathway. J Neurosci.

[38] Smith NM, Gachulincova I, Ho D, Bailey C, Bartlett CA, Norret M, Murphy J, Buckley A, Rigby PJ, House MJ, et al. An unexpected transient breakdown of the blood brain barrier triggers passage of large intravenously administered nanoparticle. Sci Rep. 2016;6. article 22595. 10.1038/srep22595 PMC4778073 26940762

[39] Fitzgerald M, Bartlett CA, Harvey AR, Dunlop SA (2010). Early events of secondary degeneration after partial optic nerve transection: an immunohistochemical study. J Neurotrauma.

[40] Miron VE, Boyd A, Zhao J-W, Yuen TJ, Ruckh JM, Shadrach JL, Pv W, Wagers AJ, Williams A, Franklin RJM, ffrench-Constant C (2013). M2 microglia and macrophages drive oligodendrocyte differentiation during CNS remyelination. Nat Neurosci.

[41] Saunders NR, Dziegielewska KM, Møllgård K, Habgood MD (2015). Markers for blood-brain barrier integrity: how appropriate is Evans blue in the twenty-first century and what are the alternatives?. Front Neurosci.

[42] Liu J, Jin X, Liu KJ, Liu W (2010). Matrix metalloproteinase-2-mediated occludin degradation and caveolin-1-mediated claudin-5 redistribution contribute to blood-brain barrier damage in early ischemic stroke stage. J Neurosci.

[43] Ryu JK, Petersen MA, Murray SG, Baeten KM, Meyer-Franke A, Chan JP, Vagena E, Bedard C, Machado MR, Coronado PER, et al: Blood coagulation protein fibrinogen promotesautoimmunity and demyelination via chemokine release and antigen presentation. Nat Commun 2015, 6. 42. 10.1038/ncomms9164 PMC4579523 26353940

[44] Chan KC, Kancherla S, Fan S-J, Wu EX: Long-term effects of neonatal hypoxia-ischemia on structural and physiological integrity of the eye and visual pathway by multimodal MRI. Invest Ophthalmol Vis Sci 2015, 56:1–9. 10.1167/iovs.14-14287 PMC4294285 25491295

[45] Lawlor M, Danesh-Meyer H, Levin LA, Davagnanam I, De Vita E, Plant GT: Glaucoma and the brain: trans-synaptic degeneration, structural change, and implications for neuroprotection. Surv Ophthalmol 2017, In Press:1–11. 10.1016/j.survophthal.2017.09.010 28986311

[46] Perego C, Fumagalli S, Zanier ER, Carlino E, Panini N, Erba E, Simoni M-GD (2016). Macrophages are essential for maintaining a M2 protective response early after ischemic brain injury. Neurobiol Dis.

[47] Leenaerts PL, Stad RK, Hall BM, Van Damme BJ, Vanrenterghem Y, Daha MR (1994). Hereditary C6 deficiency in a strain of PVG/c rats. Clin Exp Immunol.

[48] Harvey AR, Hu Y, Leaver SG, Mellough CB, Park K, Verhaagen J, Plant GW, Cui Q (2006). Gene therapy and transplantation in CNS repair: the visual system. Prog Retin Eye Res.

[49] Banks WA, Kastin AJ, Broadwell RD (1995). Passage of cytokines across the blood-brain barrier. Neuroimmunomodulation.

[50] Waguespack PJ, Banks WA, Kastin AJ (1994). Interleukin-2 does not cross the blood-brain barrier by a saturable transport system. Brain Res Bull.

[51] Kastin AJ, Akerstrom V, Pan W (2003). Interleukin-10 as a CNS therapeutic: the obstacle of the blood–brain/blood–spinal cord barrier. Mol Brain Res.

[52] Alvarez JI, Cayrol R, Prat A (2011). Disruption of central nervous system barriers in multiple sclerosis. Biochim Biophys Acta.

